# AggreCount: an unbiased image analysis tool for identifying and quantifying cellular aggregates in a spatially defined manner

**DOI:** 10.1074/jbc.RA120.015398

**Published:** 2020-10-20

**Authors:** Jacob Aaron Klickstein, Sirisha Mukkavalli, Malavika Raman

**Affiliations:** Department of Developmental Molecular and Chemical Biology, Tufts University School of Medicine, Boston, Massachusetts, USA

**Keywords:** protein quality control, proteostasis, misfolded protein, aggregate, inclusion body, stress granule, polyQ inclusion body, ubiquitin, image-based analysis, ImageJ, p97/valosin-containing protein, microscopic imaging, polyubiquitin chain, aggregation, aggresome, protein aggregation, Huntington disease, amyotrophic lateral sclerosis (ALS) (Lou Gehrig disease), protein misfolding, p97/valosin-containing protein

## Abstract

Protein quality control is maintained by a number of integrated cellular pathways that monitor the folding and functionality of the cellular proteome. Defects in these pathways lead to the accumulation of misfolded or faulty proteins that may become insoluble and aggregate over time. Protein aggregates significantly contribute to the development of a number of human diseases such as amyotrophic lateral sclerosis, Huntington's disease, and Alzheimer's disease. *In vitro*, imaging-based, cellular studies have defined key biomolecular components that recognize and clear aggregates; however, no unifying method is available to quantify cellular aggregates, limiting our ability to reproducibly and accurately quantify these structures. Here we describe an ImageJ macro called AggreCount to identify and measure protein aggregates in cells. AggreCount is designed to be intuitive, easy to use, and customizable for different types of aggregates observed in cells. Minimal experience in coding is required to utilize the script. Based on a user-defined image, AggreCount will report a number of metrics: (i) total number of cellular aggregates, (ii) percentage of cells with aggregates, (iii) aggregates per cell, (iv) area of aggregates, and (v) localization of aggregates (cytosol, perinuclear, or nuclear). A data table of aggregate information on a per cell basis, as well as a summary table, is provided for further data analysis. We demonstrate the versatility of AggreCount by analyzing a number of different cellular aggregates including aggresomes, stress granules, and inclusion bodies caused by huntingtin polyglutamine expansion.

The dynamic nature of protein folding necessitates constant surveillance to ensure the production of functional proteins (1). Errors in transcription, translation, or exposure to chemical or oxidative stress exacerbates protein misfolding (2). Protein homeostasis (proteostasis) is monitored during and after protein synthesis to promote refolding by chaperones or clearance of terminally misfolded proteins by either the ubiquitin proteasome system or autophagy (3). Components of protein quality control (PQC) work together to sense and ameliorate cellular stress caused by protein misfolding to maintain proteostasis.

Recent studies have identified an age-associated decline in the quantity, capacity, and efficiency of PQC components (4, 5). Thus, maintaining proteostasis is especially important in postmitotic cells such as neurons. This decline is often manifested by the occurrence of age-related neurodegenerative disorders, in which misfolded proteins accumulate and overwhelm proteostasis pathways (6). The formation of aggregates is observed in a wide range of neurodegenerative diseases such as Huntington's disease (7), amyotrophic lateral sclerosis (8), Parkinson's disease (9), and Alzheimer's disease (10) and contributes significantly to disease phenotypes. The morphology, localization, and composition of aggregates in neurodegenerative diseases is complex and heterogenous. Aggregates can be seeded by protein or RNA-based mechanisms and arise in distinct cellular compartments (11). Certain types of aggregates such as aggresomes and inclusion bodies are believed to be protective by sequestering misfolded proteins into a single structure for disposal (12). Furthermore, the morphology of aggregates can be quite distinct and impacts the cellular response. For example, aggregates caused by *C9orf72* gene expansions in amyotrophic lateral sclerosis form ribbon-like structures that trap proteasomes and exclude ribosomes (13). In contrast, CAG (glutamine, Q) expansions in the huntingtin gene that form polyglutamine (polyQ) aggregates in Huntington's disease are fibrillar and exclude proteasomes (14).

To study molecular mechanisms behind protein aggregation, *in vitro* models of protein aggregation have been crucial. Microscopy-based studies investigating cellular aggregates have identified numerous factors that regulate the formation and dissipation of these structures and have informed our understanding of disease (15–17). Quantification of protein aggregates has therefore become a convenient and widely accepted measure of disease phenotype severity (18). Our survey of literature relevant to this area suggests that current approaches for quantifying protein aggregates are often focused on manual image analysis (19–21). Notably, it is not always clear whether the images were analyzed in a blinded fashion (22, 23). Manual analysis of aggregates is typically limited to scoring a single cellular feature, such as, area, number, or localization of the aggregate. Such an approach is incapable of capturing multiple features at single-cell resolution from hundreds of images. Furthermore, because manual analysis often reports a population phenotype, it is unable to detect subtle differences in subpopulations of cells. Finally, manual image analysis is subjective, error-prone, and labor-intensive. An increasing number of automatic and semiautomatic image analysis software such as ImageJ and CellProfiler are available to evaluate fluorescence images (24, 25). Machine learning algorithms are also increasingly used to aid in the analysis of multiparametric, complex cellular phenotypes (26). However, incorporating various image analysis modules within these systems to create an analysis pipeline can be time-intensive and unintuitive. These disadvantages highlight a clear need for a user-friendly, flexible, high-throughput cellular image analysis method to quantify aggregates.

To improve ease, precision, speed, and reproducibility of aggregate quantification, we developed AggreCount, a novel image-processing macro, using ImageJ as a platform, to process, analyze, and quantify images. This automated tool uses common stains for nuclei, cell bodies, and aggregates as input and quantifies aggregate number, area, and cellular localization on a cell-by-cell basis. In addition to delivering an unbiased analysis of images, this level of detail provides increased analytical depth and better evaluation of subtle phenotypes that may otherwise be overlooked. All processing steps use native ImageJ plugins and functions such that additional downloads or plugins are not necessary. Furthermore, this tool is written in the ImageJ macro language, which is more accessible to those with limited programming knowledge. We have used AggreCount to analyze and quantify a variety of cellular aggregates including aggresomes, stress granules, aggresome-like induced structures (ALIS), and Htt polyQ inclusions. AggreCount will permit researchers to carry out unbiased quantification of diverse protein aggregates and is particularly suited to high-content image-based screening approaches.

## Results

### Defining settings in AggreCount for the multiparametric quantification of aggregates

**Figure 1.**
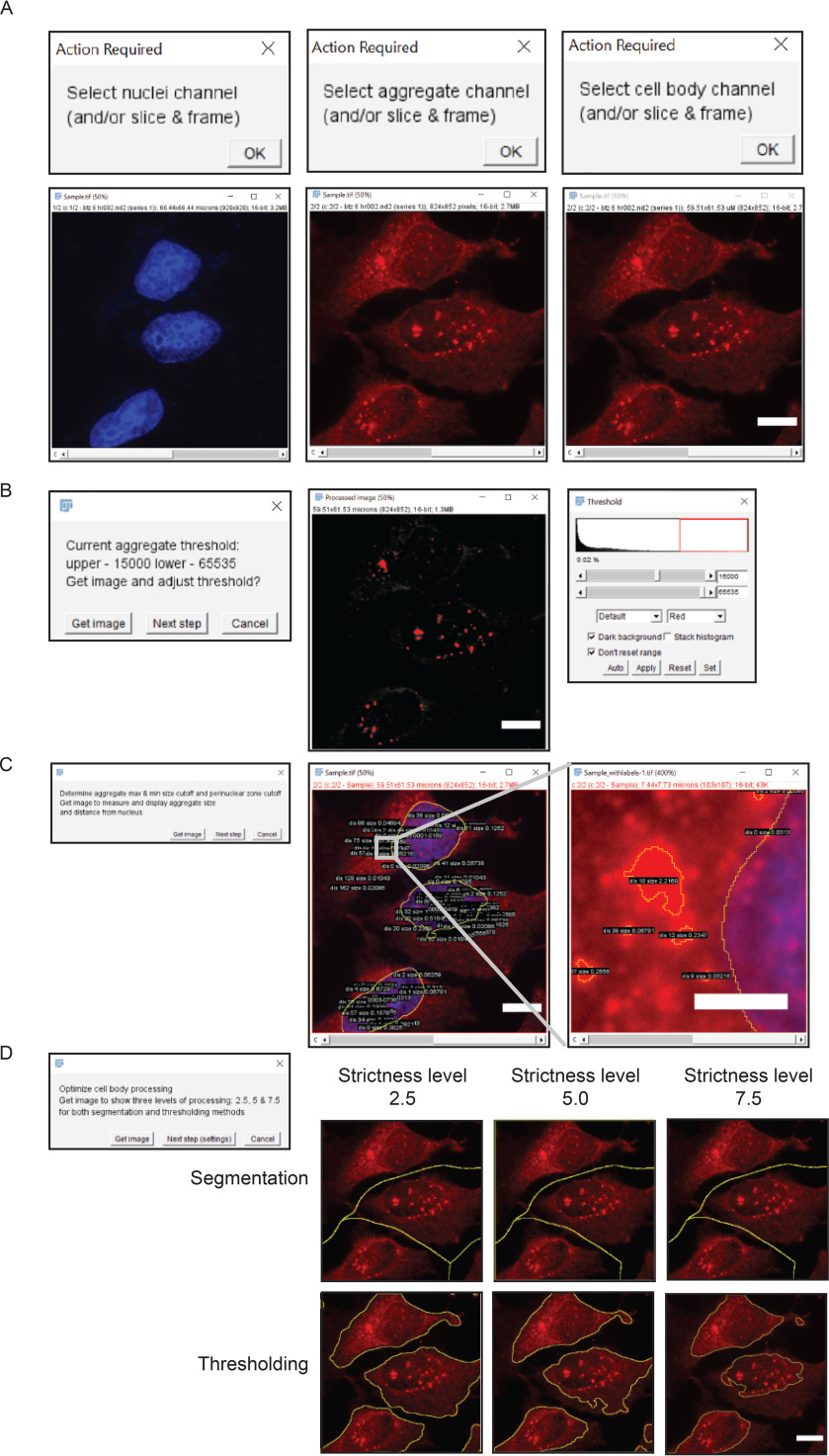
**Overview of AggreCount workflow.** *A*, user selects fluorescent channels corresponding to nuclei, aggregates, and cell bodies. *B*, threshold for aggregates is adjusted by the user through the native ImageJ threshold tool. The 0.02% value in the threshold window reflects the number of pixels in the *red box*. *C*, AggreCount displays aggregate size and distance from nucleus for the user to empirically determine the perinuclear cutoff. *D*, multiple levels of processing strictness are displayed for segmentation and thresholding of cell bodies. The user may choose any strictness level between 1 and 10. *Scale bar*, 10 μm.

The general workflow for analysis with the AggreCount macro involves three phases: assembling images, macro setup, and batch analysis. Images that will be analyzed as part of one experiment must be gathered into a single folder (the macro code and detailed instructions to run AggreCount are provided in Figs. S2 and S3). AggreCount is compatible with both common (*e.g.* TIFF, JPEG, etc.) and proprietary file formats commonly used in microscopy that are recognized by ImageJ. Each image must consist of one channel for nuclei and one for aggregate quantification. An optional third channel may be used for cell body identification. After images are compiled, the macro may be run to determine analysis settings (Fig. 1 and Fig. S1). The setup will guide the user through selecting appropriate channels for each fluorophore, adjusting the threshold, determining appropriate size cutoffs for aggregates, nuclei, and cells, as well as the best processing strictness for cell bodies. The main settings window (Fig. S1*A*) allows further refinement of the macro options. Once the variables are set, the user may proceed to batch analysis for all images in the folder. The collected data are saved as tab-delimited text files that can be imported into Excel or another data analysis software.

After selecting the folder of image files to be analyzed and selecting the “setup” option, the first image in the analysis folder will be opened. The user will be prompted to select the channel for the nuclei, aggregates, and cell bodies (Fig. 1*A*). For cell body segmentation, we recommend a dedicated channel using commercially available dyes such as CellMask; however, an antibody that detects a cytosolic protein (here we have used ubiquitin) works equally well. The user may also select the stack and frame from z-stacks or movies. AggreCount will use the selected slice and frame for that channel for every image processed. For z-stacks or movies, it may be preferable to collapse the image (*e.g.* average Z projection) before analysis for more consistent results, although in such instances the nuclear localization of aggregates should be carefully assessed. The user will then be prompted (“get image”) to select an appropriate threshold in the aggregate channel using the ImageJ threshold tool (Fig. 1*B*). AggreCount will continue to loop through the prompt to allow the user to open multiple images using the “get image” option and continue refining the threshold. It is suggested that for each analysis, at least one image with aggregates and one image without aggregates are used as positive and negative controls, respectively, to set the threshold. The final threshold parameter defined by the user will be applied to all images. Once the threshold is established, the user is prompted (“next step”) to open an image that will be processed for nuclei and aggregates using the previously established threshold. The size and distance of each aggregate from the nucleus will be displayed on the composite image (Fig. 1*C*). This allows the user to empirically determine appropriate size cutoffs for aggregates and distance limits to the perinuclear region. The final setup step allows the user to view three different levels of strictness for determining cell ROIs (levels 2.5, 5, and 7.5) as examples (Fig. 1*D*). The user may use one of these levels or any integer between 1 and 10 by entering that value into the “cell strictness” box in the main AggreCount settings window.

The main AggreCount settings window allows for manual changes to each option (Fig. S1). The values defined by the user during the setup phase will be autopopulated in the settings window. The remaining values are default values and may be changed. Perinuclear distance is the pixel distance on either side of the nuclear ROI that defines the perinuclear zone. This setting may be useful for quantification of structures such as aggresomes that reside adjacent to the nucleus. User-defined variables are available to set the aggresome, aggregate, nucleus, and cell body size minimums in microns. Additionally, there is an option to set a maximum size for aggregates. The selected channel, slice, and frame for each structure to be analyzed will be displayed and may be changed manually. If there is no channel for cell bodies or if the analysis does not require the identification of cell bodies, the user should uncheck “Find cell bodies.” The macro will calculate distances but not assign aggregates to cells.

Unchecking the “save results” option allows the macro to be run without any files being saved and may be useful when optimizing settings. Otherwise, the macro will create a new folder to save the result files. These results consist of summary text files: (i) summary analysis for all images (AC_analysis\dataset_summary.txt), (ii) all cells (AC_analysis\dataset_cells.txt), and (iii) all aggregates (AC_analysis\dataset_aggregates.txt). Additionally, a detailed results file ('FileName'_analysis.txt) and a .zip file containing cell, nuclei, and aggregate ROIs identified for each image will be saved. Finally, an explanation of each data field is saved as a text file “dataset_description” for users to reference. In batch mode, AggreCount will proceed to analyze each image in the analysis folder. Images will open in the background and will not be visible to the user. The status of the analysis may be monitored in the summary table.

### Identification and classification of aggregates

**Figure 2.**
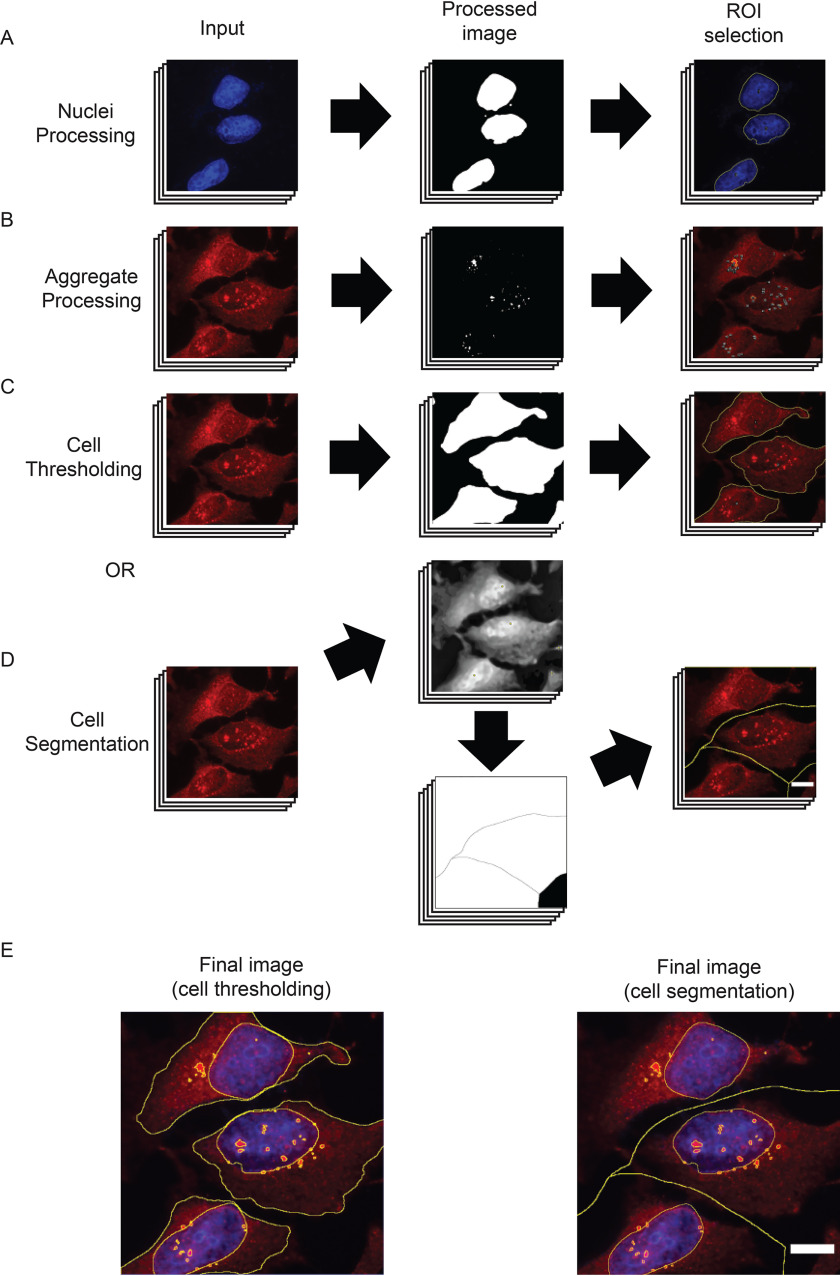
**AggreCount image-processing steps.** *A*, nuclei are processed via fluorescent signal enhancement, conversion to a binary image, and capture of ROIs. *B*, aggregates are processed via the default method or user-inserted processing steps before being converted to a binary image for ROI identification. *C*, cell thresholding via enhancement of fluorescent signal, conversion to binary image, and capture of ROIs similar to that of nuclei processing. *D*, cell segmentation process that identifies fluorescent maxima (*top middle panel*), segments cell via a watershed algorithm into a Voronoi diagram (*bottom middle panel*), which is used to capture ROIs. *E*, final merged image with nucleus, aggregate, and cell body ROIs. *Scale bar*, 10 μm.

The nuclei can be visualized using standard dyes (4′,6-diamino-2-phenylindole, Hoechst, or DRAQ5, for example), and processing of nuclei images is minimal (Fig. 2*A*). The macro uses the “enhance contrast” function before subtracting background as a function of the mean pixel intensity. The proportion of the mean that is subtracted can be modulated by changing the “strictness” setting in the settings window. Then a median filter is applied to smooth the image while maintaining borders. Properly defined borders are critical for this analysis because the nucleus ROIs define subcompartments within the cell. The image is then thresholded using the “make binary” function followed by the “dilate” and “fill holes” functions. “Dilate” is used to better define the border of the nucleus because the intensity of the fluorescent stain is often reduced at the edges of nuclei causing the outermost portion to be excluded during the “make binary” function.

The default method for processing aggregate images aims to enhance relatively small, bright puncta (Fig. 2*B*). The first step uses a crude background subtraction based on the mean pixel intensity of all areas with fluorescent signal to remove diffuse fluorescent noise. This is followed by isolating bright puncta using the difference of Gaussians approach (27). The image is then converted to 16-bit, and the threshold that was set previously is applied. This binary image is used with the “analyze particles” function to capture ROIs, which are filtered based on the previously set size criteria.

To analyze aggregates at single cell resolution, AggreCount uses two methods for cell body processing: segmentation and thresholding (Fig. 2, *C–E*). The main difference between these methods is that segmentation defines a cell by the portion of the image that contains it, whereas thresholding aims to capture the cell as a ROI without any surrounding area. Segmentation (Fig. 2*D*) is often more accurate in differentiating cells that are in close proximity, whereas thresholding (Fig. 2*C*) provides information on the cell itself such as size and pixel intensity but may omit portions of cells or erroneously merge adjacent cells. The cell area may be an important metric if treatments collapse the cytoplasm, which may spatially restrict aggregates to the perinuclear zone. AggreCount provides both methods so the user may choose the appropriate one for their images. Images of differing cell density may require optimization of segmentation parameters so testing multiple images is good practice.

Segmentation is achieved using the “find maxima” function that finds points of maximal fluorescent intensity in local areas in a merged image of the cell body and nucleus. This function utilizes a prominence setting that determines the trough required between maxima, which can be changed from the settings window using the cell body strictness option. After the maxima are determined, a pixel intensity-based watershed algorithm segments the image into a Voronoi diagram, which is used for ROI capture (Fig. 2, *D* and E). The cell thresholding method uses a process similar to that of the nuclei processing that enhances contrast, subtracts background as a proportion of the mean (that may be changed with the strictness option), and applies a median filter (Fig. 2*C*). If cell bodies are not stained, the user may deselect the “find cell bodies” option in the main settings window. The macro will proceed with localization analysis but will not be able to provide analysis on a cell-by-cell basis.

### Distance calculation and aggregate localization

**Figure 3.**
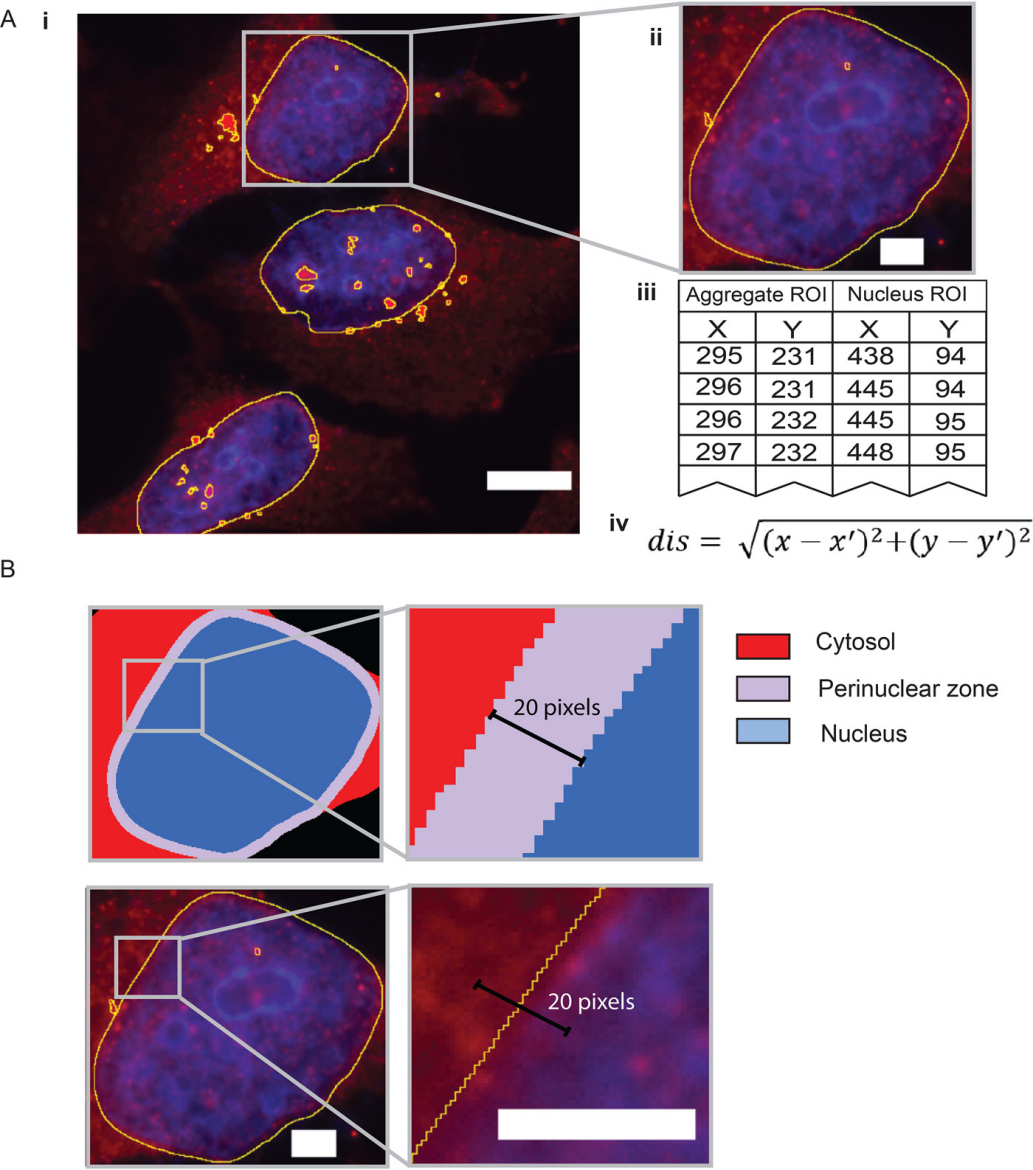
**Delineating subcellular compartments.** *A*, after ROIs have been captured (*panels i* and *ii*), each coordinate for aggregate ROIs (*panel iii*) is compared with each coordinate of nucleus ROIs to determine distance using the distance formula (*panel iv*). The smallest distance value is recorded as the aggregate distance. *B*, subcellular compartments within the cell are determined by the nucleus ROI and the user-defined distance parameter and labeled as cytosolic, perinuclear, and nuclear. The 20-pixel distance shown here denotes 10 pixels on either side of the nuclear envelope. This number should be empirically determined by the user. *Scale bar*, 10 μm in *A* (*panel i*) and 3 μm in *A* (*panel ii*) and *B*.

Once nuclei and aggregate ROIs have been captured, AggreCount will proceed to calculate the distance between aggregates and nuclei (Fig. 3*A*, *panels i* and *ii*). These distances are used to spatially classify aggregates within the cell and are calculated based on all the pixel coordinates encompassing the perimeters of the aggregate and nucleus (Fig. 3*A*, *panel iii*). The distance of each coordinate of the aggregate is calculated for each coordinate of the nucleus perimeter using the distance formula (Fig. 3*A*, *panel iv*). If the “find cell bodies” option is unchecked, aggregate distance will be calculated from the nearest nuclei. This value is used to localize the aggregate within one of three subcellular compartments: cytosol, perinuclear zone, or nucleus (Fig. 3*B*). The user defines the distance by which the perinuclear zone is determined using the perinuclear distance option in the settings window. This value is calculated on either side of the nucleus. Any aggregate that is at least partially contained in this zone is categorized as perinuclear. Thus, aggregates classified as perinuclear can reside on either side of the nuclear envelope. We have specified the default value for perinuclear distance cutoff as 10 pixels for 60× magnification images; higher values may result in cytosolic and nuclear aggregates being classified as perinuclear. The cutoff value should be empirically adjusted for images of different magnification using control images that display the desired phenotype. Aggregates that are farther inside the nucleus are considered nuclear, and aggregates that are excluded from both the perinuclear zone and the nucleus are considered cytosolic. Cell segmentation is performed using a watershed algorithm; thus, cells with complex morphology are difficult to properly segment. Therefore, aggregates in cell types with long processes such as neurons cannot be effectively classified by cellular compartment using AggreCount unless aggregates occur solely in the cell body and nucleus. As can be seen in Fig. S1*C*, AggreCount is unable to effectively segment cells with long processes such as motor neurons.

### Deploying AggreCount to quantify cellular aggregates

**Figure 4.**
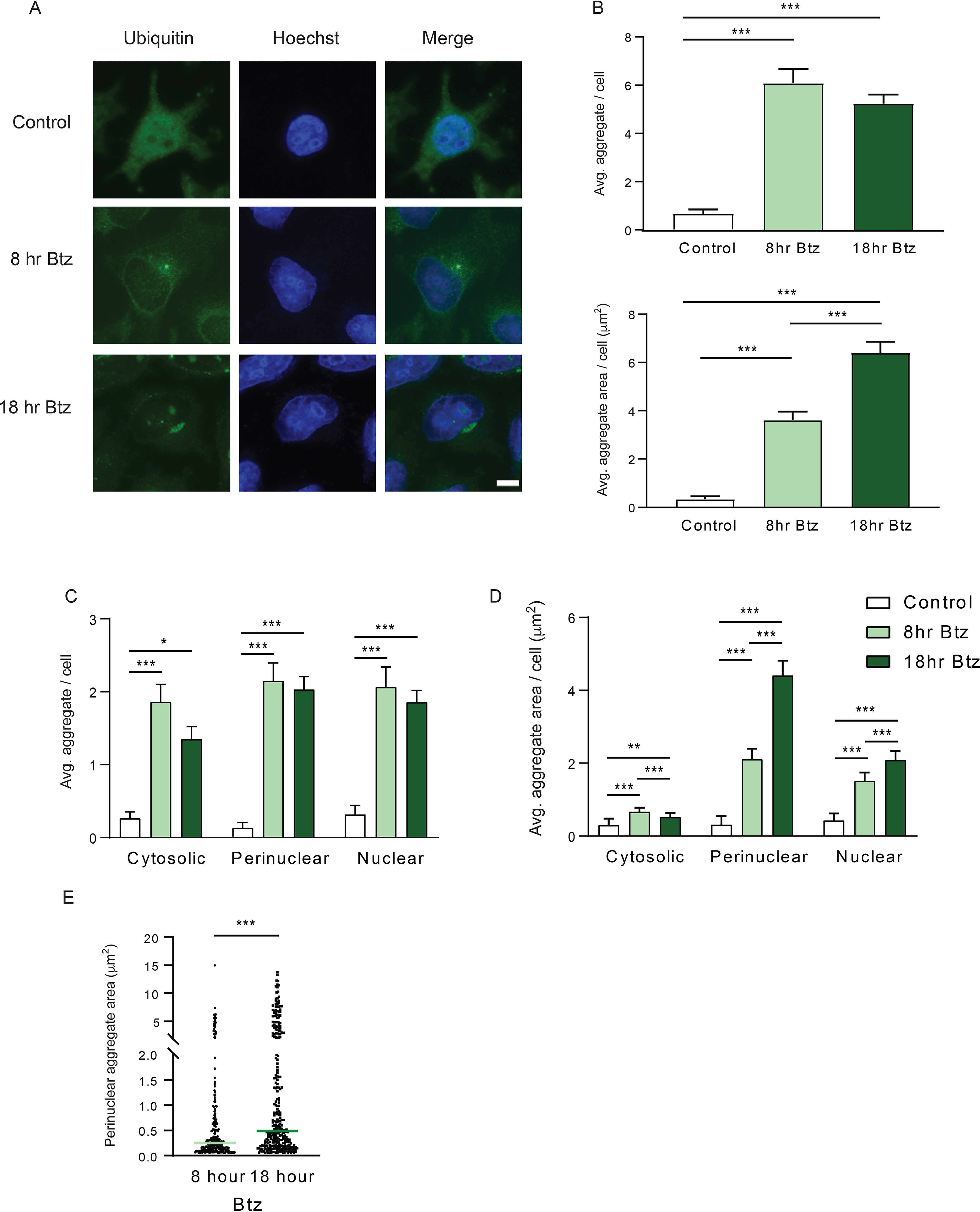
**Analysis of bortezomib-induced aggregates by AggreCount.** *A*, HeLa Flp-in TRex cells were treated with 1 μm bortezomib (*Btz*) for 8 or 18 hours (hr), fixed, and stained with an anti-ubiquitin antibody to visualize ubiquitin-positive aggregates and aggresomes. The nuclei were stained with Hoechst. *B*, AggreCount was used to determine the average (*Avg.*) number of aggregates per cell (*upper panel*) and average aggregate area (*lower panel*). *C*, the number of aggregates in different cellular compartments was quantified. *D*, the area of aggregates in different cellular compartments was determined. *E*, prolonged bortezomib treatment (18 hr) leads to an increase in perinuclear aggresome area. At least 41 cells were analyzed. The graphs show the means ± S.E. *, *p* ≤ 0.1; **, *p* ≤ 0.05; ***, *p* ≤ 0.001 as determined by one-way ANOVA with Bonferroni correction (*B–D*) or Mann–Whitney (*E*). *Scale bar*, 10 μm.

We present several examples of different cellular aggregates to demonstrate the versatility of AggreCount for their quantification. First, we quantified ubiquitin-positive aggresomes and aggregates that arise in cells upon proteasome inhibition. Aggresomes are perinuclear, membrane-delimited structures that form via retrograde trafficking of smaller cytosolic aggregates via the dynein motor (28). HeLa Flp-in TRex cells were treated with the reversible proteasome inhibitor bortezomib for 8 or 18 hr before being fixed and stained for ubiquitin and nuclei (Hoechst) (Fig. 4*A*). Images were analyzed using AggreCount. As shown in Fig. 4*B*, both time points have an increased number and size of aggregates compared with the untreated controls. The AggreCount analysis allows for the stratification of aggregates by subcellular location at single cell resolution. This deeper analysis reveals that the 18 hr treatment reduces the number of cytosolic aggregates while the number of perinuclear and nuclear aggregates remain stable (Fig. 4*C*). However, the size of the perinuclear aggregates greatly increases (Fig. 4, *D* and E).

We asked how AggreCount compared with other methods used for aggregate quantification. As mentioned above, existing methods primarily involve manual analysis; therefore we determined how long it took for an experienced ImageJ user to manually analyze the same set of images as AggreCount. In addition, we also used an existing CellProfiler pipeline that counts cellular speckles. We found no significant difference in the findings between these two methods and AggreCount for analyzing the number of aggregates per cell (Fig. S4*A*). However, manual quantification required over an hour for image analysis, whereas both AggreCount and CellProfiler were able to analyze the same set of images in ∼90 s. Importantly, manual quantification and CellProfiler do not provide any information pertaining to the subcellular localization of aggregates. Thus, a significant advantage of AggreCount is the ability to spatially localize aggregates in a facile manner. Although sample preparation and good imaging practices yield the most robust analysis (see “Experimental procedures”), we find that the difference of Gaussians method used for thresholding aggregates enables AggreCount to be adept at overcoming image thresholding challenges such as uneven background illumination and image saturation (Fig. S4, *B* and *C*).

**Figure 5.**
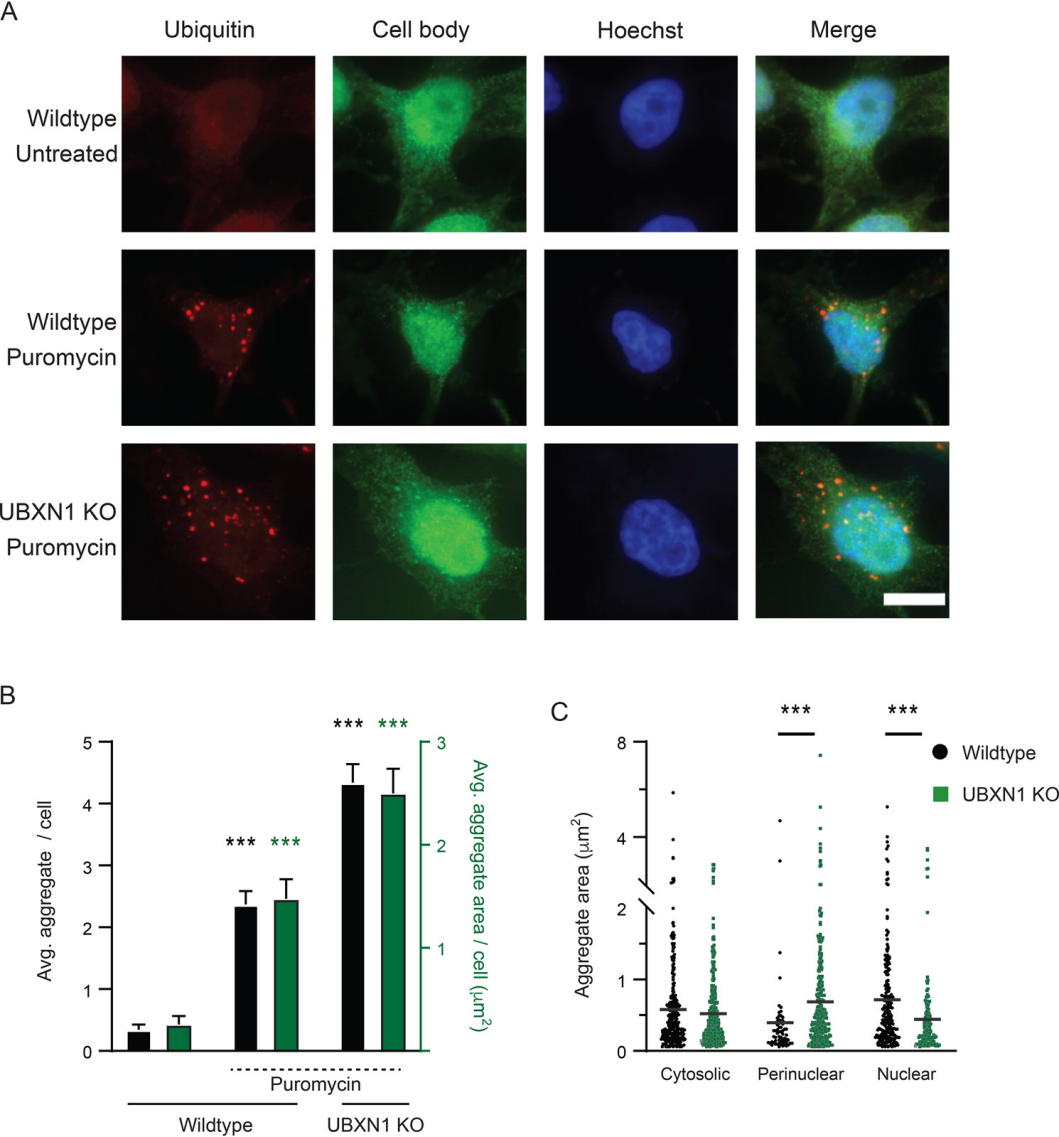
**ALIS quantification by AggreCount.** *A*, HeLa Flp-in TRex cells (WT and UBXN1 KO) were treated with 5 µg/ml of puromycin for 2 hr, fixed, and stained with an anti-ubiquitin antibody to visualize ubiquitin-positive ALIS. The nuclei were stained with Hoechst. *B*, AggreCount was used to determine the average number of ALIS per cell (*black bars*) and average ALIS area (*green bars*). UBXN1 KO cells have a greater number of ALIS with increased areas *C*, the area of ALIS in different cellular compartments was determined. At least 96 cells were analyzed. The graphs show the means ± S.E. ***, *p* ≤ 0.001 as determined by one-way ANOVA with Bonferroni correction (*B*) or Kruskal–Wallis test with Dunn correction (*C*). In *B*, significance is calculated with respect to WT untreated for average aggregate per cell (*black*) and aggregate area/cell (*green*). *Scale bar*, 10 μm.

Puromycin treatment causes premature chain termination during translation and the accumulation of defective ribosomal products (29, 30). Defective ribosomal products are frequently sequestered into punctate, ubiquitin-positive structures in the cytosol referred to as ALIS (16, 31). AggreCount was able to effectively identify ALIS in puromycin-treated cells (Fig. 5*A*). We have previously shown that ubiquitin X domain containing 1 (UBXN1) is required for the clearance of ALIS (32). The increase in ALIS in UBXN1 KO cells was effectively captured by AggreCount (Fig. 5, *A–C*). Notably, ALIS in UBXN1 KO cells were increased in the perinuclear area relative to WT cells (Fig. 5*C*), illustrating capability of AggreCount to spatially localize aggregates when perturbations alter the subcellular localization of aggregates.

**Figure 6.**
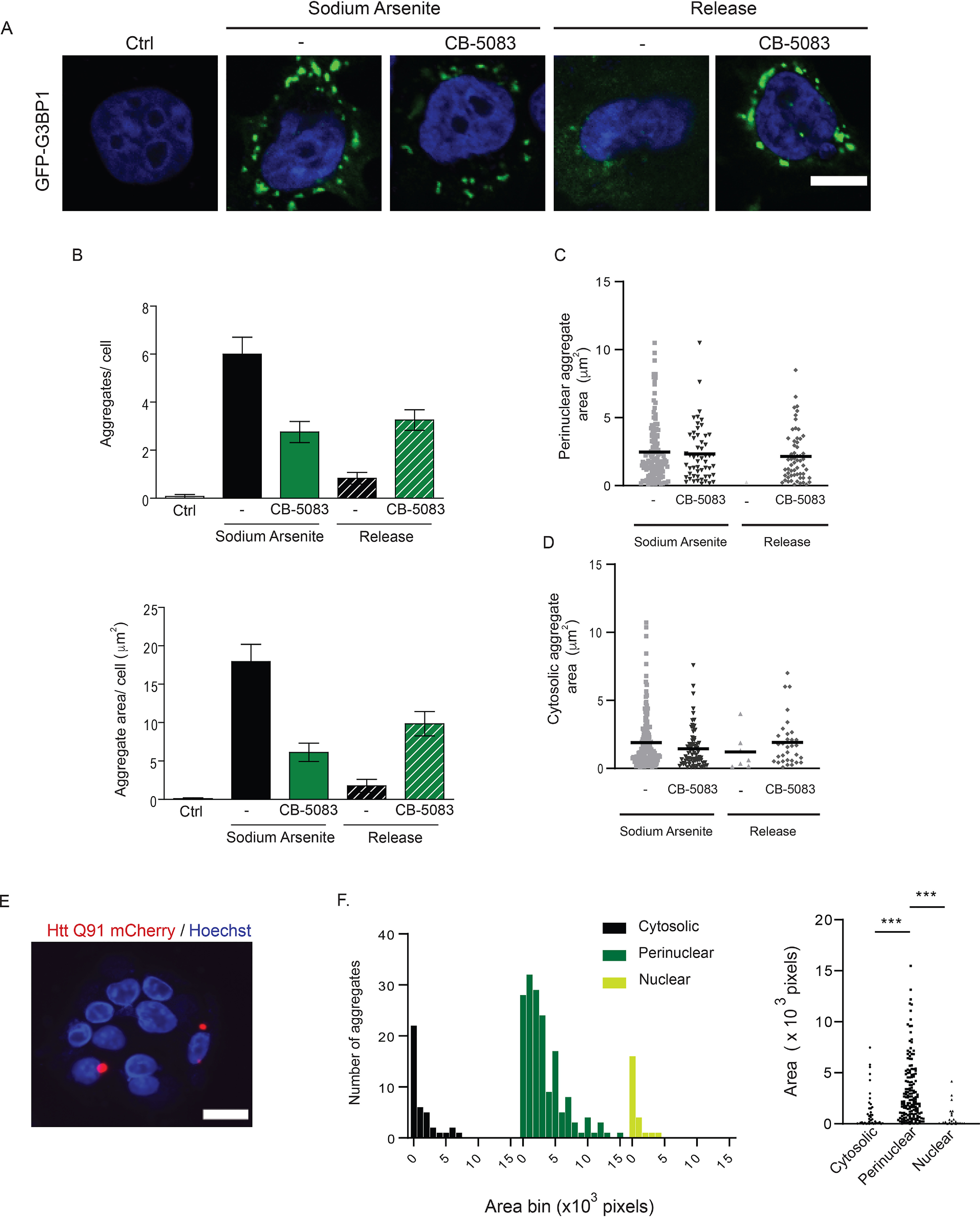
**Analysis of stress granules and inclusion bodies by AggreCount.** *A*, HeLa Flp-in TRex cells stably expressing the stress granule marker GFP-G3BP1 were treated with 0.5 mm sodium arsenite for 1 hr. The cells were pretreated with 5 μm CB-5083 (p97 inhibitor) for 1 hr prior to addition of 0.5 mm sodium arsenite for 1 hr. The cells were released for 4 hr into drug-free medium or in medium containing CB-5083, fixed, and imaged. The nuclei were stained with Hoechst. *B*, AggreCount was used to determine the average number of stress granules per cell (*upper graph*) and average stress granule area (*lower graph*). *C* and *D*, the area of perinuclear (*C*) and cytosolic stress granules (*D*) was quantified. *E*, U2OS Htt91-mCherry cells were induced with 1 µg/ml of doxycycline for 48 hr. The cells were fixed and imaged for inclusion bodes. The nuclei were stained with Hoechst. *F*, *left panel*, a histogram of inclusion body areas based on their cellular location was quantified and binned based on the indicated size bins. *Right panel*, the size distribution of individual inclusion bodies by cellular location. At least 13 cells were analyzed for *B–D*, and at least 1036 cells were analyzed for *E* and *F*. The graphs show the means ± S.E. ***, *p* ≤ 0.001 as determined by one-way ANOVA with Kruskal–Wallis test with Dunn correction. *Scale bar*, 10 μm for *A* and *E*. *Ctrl*, control.

We next addressed RNA-based cellular aggregates such as stress granules. Stress granules form as a result of a variety of cellular stressors that cause stalling of translation and disassembly of ribosomes (33). Stress granules contain 40S subunits of ribosomes, ribonucleoproteins, and RNAs, and their inappropriate persistence in neurons is linked to neurodegeneration (34). We induced stress granule formation in HeLa cells stably expressing GFP-tagged G3BP1 (a *bona fide* stress granule component), using sodium arsenite, a well-established chemical that robustly induces stress granule formation (Fig. 6*A*). Release of cells post-sodium arsenite treatment into drug-free medium leads to the dissolution of stress granules (Fig. 6, *A* and *B*). Previous studies have demonstrated a role for p97, an essential, ubiquitin-selective, AAA ATPase that is critical for multiple PQC processes in the cell, in the clearance of stress granules (35–37). Treatment of cells with an ATP-competitive p97 small molecule inhibitor (CB-5083) during the formation or release periods produced distinct outcomes (38). Co-treatment of cells with both sodium arsenite and CB-5083 results in the formation of fewer and smaller stress granules compared with sodium arsenite treatment alone (Fig. 6, *A* and *B*). Conversely, p97 inhibition during the release period prevents the clearance of stress granules predominantly in the perinuclear region (Fig. 6, *A–D*).

Expansion of a CAG tract in the first exon of the huntingtin (*Htt*) gene beyond a threshold of ∼35–40 repeats causes Huntington's disease and results in a mutant Htt protein containing an expanded polyQ segment (39). Expression of exon 1 Htt fragments longer than 40 residues leads to the formation of insoluble amyloid-like aggregates of Htt known as inclusion bodies and causes neurodegeneration (41). We measured inclusion body formation using a U2OS cell line stably expressing a doxycycline-inducible Htt polyQ91 construct tagged with mCherry (40). AggreCount was able to identify polyQ aggregates in cells and classify their localization as predominantly perinuclear as previously reported (Fig. 6, *E* and *F*) (41). Previous studies have suggested that large inclusion bodies formed by polyQ are largely cytoprotective and smaller soluble aggregates are cytotoxic (42). Because AggreCount can effectively identify and quantify the area of cytosolic and perinuclear aggregates (Fig. 6*F*); conditions that shift the dynamic between cytosolic aggregates and inclusion bodies can be rapidly assessed using AggreCount.

## Discussion

ImageJ and CellProfiler are two of the most commonly used software for immunofluorescent image analysis. Both provide a robust set of tools for image processing, thresholding, and capture of data (43). Our survey of literature pertaining to image analysis of aggregates found no uniform method of image analysis. In many cases, aggregates were quantified manually, which is time-intensive and introduces bias and human error. CellProfiler allows for the batch processing and analysis of images, which helps alleviate these problems. However, there does not currently exist a pipeline for aggregate quantification and localization. Additionally, CellProfiler is limited by its available modules, whereas AggreCount may utilize any native ImageJ tool or user-created plugin. Finally, a moderate level of image-processing background is required for initial development of CellProfiler pipelines (24). Here, we present the AggreCount macro that is based on the widely used FIJI distribution of ImageJ (44). This macro provides an unbiased automated platform for aggregate quantification on a cell-by-cell basis. Unlike other available image analysis tools, AggreCount may be used directly out of the box without any modifications for a wide variety of immunohistochemical stains providing in-depth aggregate quantification. We have deposited AggreCount to GitHub (https://aggrecount.github.io/) to be downloaded as a macro for the reproducible and efficient analysis of aggregates by the research community. Although we believe our method for processing images with aggregates works well for a wide variety of applications, we understand that these processing steps may not be applicable for all experiments. Thus, the portions of the macro that contain the processing steps for aggregates, nuclei, and cells are highlighted and may be altered by the user (Fig. S1*B*). The ImageJ macro language was designed such that users with limited programming knowledge may utilize it. The “record” function in ImageJ may be used to further customize the script in a user-specific manner.

We demonstrate that AggreCount can capture and quantify a variety of distinct cellular aggregates that arise because of specific perturbations, including aggresomes, ALIS, stress granules, and Htt inclusion bodies. Although the AggreCount macro was designed for the quantification of aggregates, it may be used to quantify and localize organelles, such as mitochondria or lysosomes, and disease markers in neurodegeneration, such as TDP-43 or FUS that relocalize from the nucleus to the cytoplasm and aggregate. Additionally, because this tool analyzes ROIs on a cell-by-cell basis, it may be used to parse out different cell types in a heterogenous population provided individual cell populations can be uniquely labeled. The AggreCount macro provides a powerful platform for unbiased, automated quantification and localization of cellular aggregates.

## Experimental procedures

### Antibodies and chemicals

The mouse anti-ubiquitin (FK2) used for immunofluorescence was from EMD Millipore. Hoechst dye was from Sigma, and Alexa Fluor–conjugated secondary antibodies were from Molecular Probes. Bortezomib was obtained from Selleckchem. Sodium arsenite and puromycin were from Sigma.

### Cell culture

HeLa Flp-in TRex (kind gift from Brian Raught, University of Toronto), HeLa Flp-in TRex GFP-G3BP1 and U2OS HttQ91-mCherry (kind gift from Ron Kopito, Stanford University) were cultured in Dulbecco's modified Eagle's medium supplemented with 10% fetal bovine serum and 100 units/ml penicillin. The cells were maintained in a humidified, 5% CO_2_ atmosphere at 37 ˚C. The cells were treated with 1 μm bortezomib, 0.5 mm sodium arsenite, 5 μm CB-5083, and 5 µg/ml of puromycin for the indicated times. HttQ91-mCherry expression was induced by treating cells with 1 µg/ml of doxycycline for 48 hr. UBXN1 knockout cells were previously described (34). GFP-G3BP1 stable HeLa Flp-in TREX cell lines were generated by lentiviral transduction as previously reported (45). Cells expressing low levels of GFP-G3BP1 were isolated by flow cytometry. Care should be taken to determine whether treatment of cells with specific agents causes the cytosol to collapse or shrink. In such cases, cytosolic aggregates may be overwhelmingly identified as perinuclear. In such cases, it is useful to use CellMask and the threshold tool to measure the area of cells between treatment conditions.

### Immunofluorescence and microscopy

HeLa Flp-in T-REX and U2OS cells were grown on coverslips (no. 1.5) in a 12-well plate. The cells were washed briefly in PBS and fixed with either 4% paraformaldehyde at room temperature (puromycin treatment) for 15 min or ice-cold methanol at 4˚C for 10 min. The cells were washed three times in PBS and then blocked in 2% BSA, 0.3% Triton X-100 in PBS for 1 hr. For the puromycin treatment, the cells were blocked in 3% chicken serum plus 0.1% Triton X-100 in PBS for 1 hr. The coverslips were incubated with the indicated antibodies overnight at 4 ˚C in a humidified chamber, washed, and incubated for 1 hr with the appropriate Alexa Fluor–conjugated secondary antibodies for 1 hr in the dark at room temperature. The cells were washed with PBS, and the nuclei were stained with Hoechst dye and mounted onto slides. Images were collected by using a Nikon A1R scan head with a spectral detector and resonant scanners on a Ti-E motorized inverted microscope equipped with a 60× Plan Apo 1.4–numerical aperture objective lens. The indicated fluorophores were excited with a 405-, 488-, or 594-nm laser line.

For robust image analysis and quantification, standardized sample preparation and image acquisition practices must be followed. Culture conditions must be optimized and observed regularly. For optimal image analysis, we recommend imaging low to moderate cell densities as images with cells in close proximity to one another negatively impact accurate cell counts and segmentation. Imaging must be carried out on the same microscope at identical magnifications and exposure or laser settings because they will heavily influence the outcome of thresholding methods. All the images must be adequately focused using nuclear or fluorophore staining. Additionally, imaging is recommended to capture images in the nuclear stain channel to remove potential unconscious bias in data selection.

### Alternative quantification methods

Manual analysis was performed by isolating individual cells from images, applying a threshold, and utilizing the analyze particles ImageJ function. CellProfiler analysis used the publicly available Speckle Counter pipeline with adjustments for file names, image processing, and thresholding.

## Data availability

The AggreCount macro is available in the supporting information associated with this article and will be uploaded to GitHub (https://aggrecount.github.io/).

## Supplementary Material

Supporting Information
