# Supplementary material for: AggreCount: an unbiased image analysis tool for identifying and quantifying cellular aggregates in a spatially defined manner
Source: J Biol Chem. 2020 Oct 20;295(51):17672–83. doi: 10.1074/jbc.RA120.015398 (PMC7762942; doi:10.1074/jbc.RA120.015398)
Supplement: Supporting Information [file supp_295_51_17672__index.html]

AggreCount: An unbiased image analysis tool for identifying and quantifying cellular aggregates in a spatially-defined manner — Method to quantify cellular aggregates — AggreCount: an unbiased image analysis tool for identifying and quantifying cellular aggregates in a spatially defined manner — Method to quantify cellular aggregates — Supporting Information 

# AggreCount: an unbiased image analysis tool for identifying and quantifying cellular aggregates in a spatially defined manner

## Supporting Information

- Supporting Information (to be published online) - Supporting Information Legends
