# Supplementary material for: AggreCount: an unbiased image analysis tool for identifying and quantifying cellular aggregates in a spatially defined manner
Source: J Biol Chem. 2020 Oct 20;295(51):17672–83. doi: 10.1074/jbc.RA120.015398 (PMC7762942; doi:10.1074/jbc.RA120.015398)

AggreCount: An unbiased image analysis tool for identifying and quantifying cellular aggregates in a spatially-defined manner

Jacob Aaron Klickstein, Sirisha Mukkavalli and Malavika Raman

Supporting information Legends

Supporting Information 1. Settings and aggregate processing may be customized by the user. **A.** The main dialogue window may be used to change analysis parameters in a user-friendly manner. **B.** Processing steps have been highlighted within the macro code for easy editing. **C.** Cells with processes or other complex morphologies cannot be segmented by AggreCount. Motor neurons were stained with Tuj1 (green) and Hoechst (blue). AggreCount was used for thresholding and segmentation.

Supporting Information 2. Code for AggreCount

Supporting Information 3. Step by step instruction for running AggreCount. Sample images to can be found at the following link : https://tufts.box.com/s/ys3kktb5ujdnilyqu8mnup3mrtiej6b6

Supporting Information 4. Comparison of AggreCount with other analysis methods. **A**. Comparison of AggreCount against manual quantification and CellProfiler's Speckle Counting pipeline. A representative image of a processed cell with threshold applied by all three methodologies (i). Quantification of cell counts (ii) and aggregates per cell (iii) for each methodology using the test images provided in the Box link above. **B.** AggreCount's processing method eliminates uneven background illumination allowing for the capture of aggregates in poor quality images. Sample image is shown before processing (right panels), after processing (middle panels), and after ROI detection and capture (left panels). **C.** AggreCount reliably quantifies aggregates in images of different exposures. Images of the same field were captured at four different exposures: 2.5 seconds (i), 12 seconds (ii), 25 seconds (iii), and 50 seconds (iv). Each image was quantified for cell counts, cells with aggregates, and cells with aggresomes (v). Additionally, number of aggregates, aggregate area, and aggregate size per cell (vi) were quantified.

Supporting information 1

A

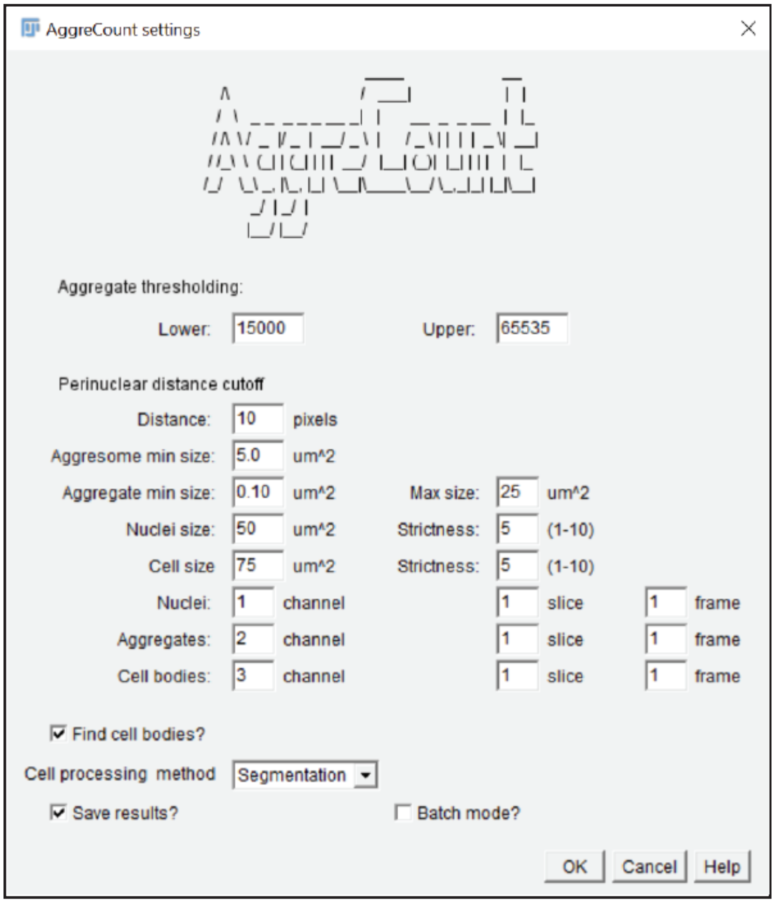

B

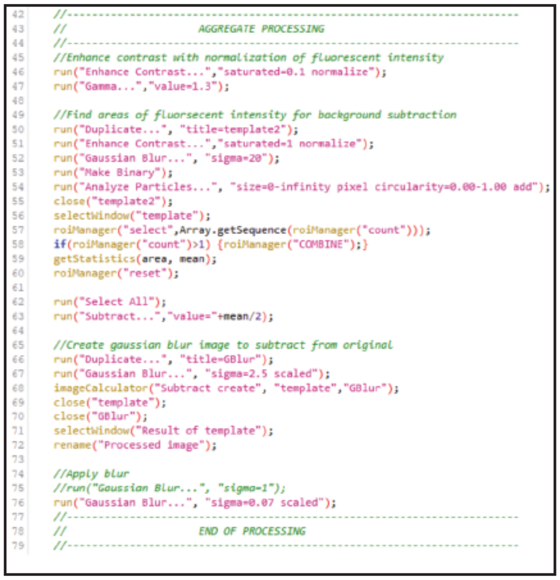

```
//--------------------------------------------------------------------------------
//                          AGGREGATE PROCESSING
//--------------------------------------------------------------------------------
//Enhance contrast with normalization of fluorescent intensity
run("Enhance Contrast...","saturated=0.1 normalize");
run("Gamma...","value=1.3");

//Find areas of fluorsecent intensity for background subtraction
run("Duplicate...", "title=template2");
run("Enhance Contrast...","saturated=1 normalize");
run("Gaussian Blur...", "sigma=20");
run("Make Binary");
run("Analyze Particles...", "size=0-infinity pixel circularity=0.00-1.00 add");
close("template2");
selectWindow("template");
roiManager("select",Array.getSequence(roiManager("count")));
if(roiManager("count")>1) {roiManager("COMBINE");}
getStatistics(area, mean);
roiManager("reset");

run("Select All");
run("Subtract...","value="+mean/2);

//Create gaussian blur image to subtract from original
run("Duplicate...", "title=GBlur");
run("Gaussian Blur...", "sigma=2.5 scaled");
imageCalculator("Subtract create", "template","GBlur");
close("template");
close("GBlur");
selectWindow("Result of template");
rename("Processed image");

//Apply blur
//run("Gaussian Blur...", "sigma=1");
run("Gaussian Blur...", "sigma=0.07 scaled");
//--------------------------------------------------------------------------------
//                          END OF PROCESSING
//--------------------------------------------------------------------------------
```

C

Tuj1 / Hoechst

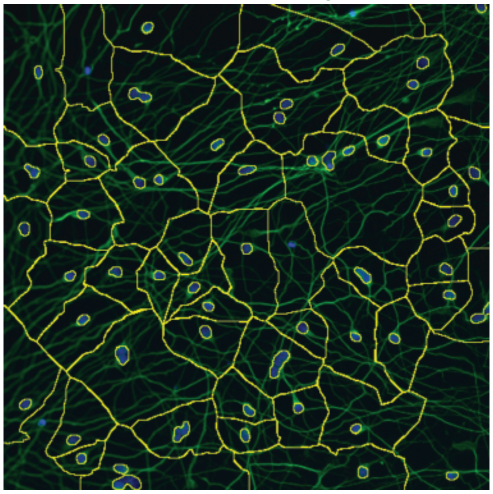

```
//------------------------------------------------------------------------
//                  Aggrecount v1.1 2020
//------------------------------------------------------------------------
/*
 * Welcome to the Aggrecount Macro developed by the Raman Lab at Tufts University, Boston, MA
 * This macro is intended to quantify immunofluorescent images of cultured cells stained for
 * the nucleus (eg dapi), aggregates (eg ubiquitin), and cell body (eg Cell Mask). Each image should
 * be a multi-channel or stacked image with each of these stains.
 *
 * This macro may be modified to fit your needs by editing the processing steps below. Image processing
 * may use any Fiji plugin or function that has been adapted for use in macros. The processing must end
 * with a binary image for nuclei, aggregates and the cell thresholding method. Cell segmentation may
 * take 8-bit or 16-bit images and requires that the Fiji function "Find Maxima..." is run with the
 * "segmentation" option be selected.
 *
 * Patch notes
 * 1.1  - Fixed indexing error when an aggregate ROI happens to lie between two cell ROIs
 *      - Added "Largest perinuclear aggregate size" to dataset_cells for post-hoc aggresome analysis
 *                      - Changed DoG blur to scale with image scale
 *      - Changed final gaussian blur of aggregates to scale with image scale
 *      - Added a gamma process step to enhance bright puncta
 *      - Added support for multi-channel stacks using duplicate function
 *      - Added additional instructions in setup prompts for clarity
 *      - Added "dataset_description" save file with explanations for each data field
 *      - Fixed error when there is a folder name < 4 characters in the analysis folder
 *      - Added "help" button to main dialog window that directs to BioRx preprint of the AggreCount
article
 */
```

```
requires("1.52p")

suffixes =
newArray("1sc","4etc","afi","aim","al3d","ali","am","amiramesh","apl","arf","avi","bip","bmp","btf","c0
1","ch5","cif","cr2","crw","csv","dat","dcm","dib","dicom","dm2","dm3","dm4","dv","eps","epsi","exp",
"fff","fits","flex","fli","frm","gel","gif","grey","gz","h5","hdr","hed","his","htd","hx","i2i","ics","ids","im3
","img","ims","inr","ipl","ipm","ipw","j2k","jp2","jpf","jpg","jpk","jpx","klb","l2d","labels","lei","lif","liff",
"lim","map","mea","mnc","mng","mod","mrc","mrcs","mrw","msr","mtb","naf","nd","nd2","ndpi","ndpi
s","nef","nhdr","nii","nrrd","obf","obsep","oib","oif","oir","ome","par","pcoraw","pcx","pds","pic","png
","pnl","ps","r3d","raw","rcpnl","rec","res","scn","sdt","seq","sif","sld","spc","st","stk","stp","svs","sxm"
,"tf2","tf8","tif","tiff","tnb","top","txt","v","vff","vms","vsi","wpi","xdce","xml","xv");



function AggregateROI(ubq,dapiroi,aggroi,setup,lower,upper,aggcutmin,aggcutmax,batch)
{
	selectWindow(ubq);
	run("Select All");
	run("Duplicate...", "title=template");
	run("16-bit");



	//---------------------------------------------------------------------
	//			AGGREGATE PROCESSING
	//---------------------------------------------------------------------
	//Enhance contrast with normalization of fluorescent intensity
	run("Enhance Contrast...","saturated=0.1 normalize");
	run("Gamma...","value=1.3");

	//Find areas of fluorsecent intensity for background subtraction
	run("Duplicate...", "title=template2");
	run("Enhance Contrast...","saturated=1 normalize");
	run("Gaussian Blur...", "sigma=20");
```

```
run("Make Binary");
run("Analyze Particles...", "size=0-infinity pixel circularity=0.00-1.00 add");
close("template2");
selectWindow("template");
roiManager("select",Array.getSequence(roiManager("count")));
if(roiManager("count")>1) {roiManager("COMBINE");}
getStatistics(area, mean);
roiManager("reset");

run("Select All");
run("Subtract...","value="+mean/2);

//Create gaussian blur image to subtract from original
run("Duplicate...", "title=GBlur");
run("Gaussian Blur...", "sigma=2.5 scaled");
imageCalculator("Subtract create", "template","GBlur");
close("template");
close("GBlur");
selectWindow("Result of template");
rename("Processed image");

//Apply blur
//run("Gaussian Blur...", "sigma=1");
run("Gaussian Blur...", "sigma=0.07 scaled");
//----------------------------------------------------------------------
//                  END OF PROCESSING
//----------------------------------------------------------------------
```

```
	//Allow manual manipulation of threshold during threshold setup
	if(setup)
	{
		run("Grays");
		run("Threshold...");
		setThreshold(lower,upper);
		aggnum = 0;
		waitForUser("Adjust threshold in thresholding window\nClick 'OK' when satisfied");
	}
	//Apply threshold automatically after setup
	else
	{
		setThreshold(lower,upper,"black&white");

		run("Convert to Mask");
		run("Analyze Particles...", "size="+aggcutmin+"-"+aggcutmax+" circularity=0.00-1.00
add");
		close("Processed image");

		//Get number of aggregate ROI's and save them
		aggnum = roiManager("Count");
		if(aggnum>0) {roiManager("save", aggroi);}

		//Display nuclei ROI's if batch mode is not selected
		selectWindow(ubq);
		if(batch==false)
		{
			roiManager("Show All without labels");
			roiManager("Measure");
```

```
                    waitForUser("Aggregate ROIs");
                    run("Clear Results");
                }

                roiManager("reset");
        }

        return aggnum;
}

function NucleiROI(dapi,dapiroi,nuccut,nucstrictness,batch)
{
        selectWindow(dapi);
        run("Select All");
        run("Duplicate...", "title=template");
        //-----------------------------------------------------------------------
        //              NUCLEI PROCESSING
        //-----------------------------------------------------------------------

        //Enhance contrast
        run("Enhance Contrast...", "saturated=1 normalize");

        //Subtracting background as a proportion of the mean
        getStatistics(area, mean, min, max, std, histogram);
        run("Subtract...","value="+(mean/(11-nucstrictness)));

        //Median filter to blur and preserve edges
        run("Median...", "radius=13");
```

```
//Threshold and turn into binary image
run("Make Binary");
run("Dilate");
run("Fill Holes");

//----------------------------------------------------------------------
//                END OF PROCESSING
//----------------------------------------------------------------------

run("Analyze Particles...", "size="+nuccut+"-infinity circularity=0.00-1.00 add");
close("template");

//Display nuclei ROI's if batch mode is not selected
selectWindow(dapi);
if(batch==false)
{
	roiManager("Show All without labels");
	roiManager("Measure");
	waitForUser("Nuclei ROIs");
	run("Clear Results");
}

//Get average area of nuclei ROI
roiManager("select",Array.getSequence(roiManager("count")));
if(roiManager("count")>1) {roiManager("COMBINE");}
getStatistics(area);
nucarea = area/roiManager("Count");

//Save ROI's temporarily
```

```
	roiManager("save", dapiroi);
	roiManager("reset")

	return nucarea;
}

function cellROI(cell,dapiroi,aggroi,cellroi,cellcut,nucarea,batch,segmentation,strictness)
{
	selectWindow(cell);
	run("Select All");
	run("Duplicate...","title=template");

	if(segmentation) //For cell segmentation
	{
		//----------------------------------------------------------------------
		//			CELL SEGMENTATION
		//----------------------------------------------------------------------

		//create a composite image of nuclei and cell bodies
		run("Merge Channels...", "c1="+dapi+" c2="+cell+" create keep");
		run("Stack to RGB");
		run("16-bit");
		//Enhance contrast
		run("Enhance Local Contrast (CLAHE)", "blocksize=249 histogram=239 maximum=18 
mask=*None* fast_(less_accurate)");
		//Subtract background with rolling ball algorithm
		run("Subtract Background...", "rolling=300");
		//Apply gaussian filter to blur
		run("Gaussian Blur...", "sigma=3 scaled");
```

```
		//Find local maxima and apply pixel intensity watershed algorithm
		run("Find Maxima...", "prominence="+(strictness*4)+" strict exclude 
output=[Segmented Particles]");


		//-----------------------------------------------------------------------
		//			END CELL SEGMENTATION
		//-----------------------------------------------------------------------
	}
	else
	{
		//-----------------------------------------------------------------------
		//			CELL THRESHOLDING
		//-----------------------------------------------------------------------
		//Enhance contrast
		run("Enhance Contrast...", "saturated=10 normalize");
		//Subtract proportion of mean pixel intensity
		getStatistics(area, mean);
		run("Subtract...","value="+(mean*(strictness/5)));
		//Apply median filter to blur and preserve edges
		run("Median...", "radius=20");

		//Turn into binary image and fill in nuclei ROIs
		run("Make Binary");
		roiManager("Open",dapiroi);
		roiManager("Select",Array.getSequence(roiManager("Count")));
		Roi.setFillColor("white");
		roiManager("Fill");
		roiManager("Reset");
```

```
		//Turn into binary image and dilate
		run("Make Binary");
		run("Dilate");

		//----------------------------------------------------------------------
		//			END CELL THRESHOLDING
		//----------------------------------------------------------------------
}

run("Analyze Particles...", "size="+cellcut+"-inifinty pixel circularity=0.00-1.00 add");
if(segmentation) {close("Composite*");}
close("template");


//Cycles through each cell ROI and if it does not contain at least 1/5 of a nuclei ROI, delete it
selectWindow(cell);
rawcellnum = roiManager("count");
roiManager("Open",dapiroi);
totalroinum = roiManager("count");
roi=0;
for(j=0;j<rawcellnum;j++)
{
		containsnuc = false;
		for(k=rawcellnum;k<totalroinum;k++)
		{
				tempdapinum = roiManager("Count") - (totalroinum-k);
				roiManager("select",newArray(tempdapinum,roi));
				roiManager("AND");
				if(selectionType>-1)
```

```
        {
                getStatistics(narea, nmean, min, max, nstd);
                if(narea>nucarea/5)
                        {
                                containsnuc = true;
                                k = totalroinum;
                        }
        }
}

/*The code below contains a filter for only selecting cells that have positive staining
 * in the aggregate channel. Cells without staining in the aggregate channel will be
deleted*/

//selectWindow(ubq);
//roiManager("Select",roi);
//getStatistics(area, mean, min, max, std, histo);
//for(k=0;k<lengthOf(histo);k++)
//{
//        histo[k] = histo[k] * k * (max-min)/256;
//}
//histo = Array.deleteValue(histo,0);
//histo = Array.slice(histo,round(lengthOf(histo)*19/20),lengthOf(histo));
//Array.getStatistics(histo, min, max, mean);
//if(mean<1000) {containsnuc=false;}


roiManager("deselect");
if(containsnuc==false)
```

```
		{
			roiManager("Select",roi);
			roiManager("Delete");
			roi = roi - 1;
		}
		roi = roi + 1;
	}

	//Removes nuclei ROI from ROI manager
	roiManager("Select",Array.slice(Array.getSequence(roiManager("Count")),roi,roiManager("Coun
t")));
	roiManager("Delete");

	//Displays cell ROI's if batch mode is not selected
	if(batch==false)
	{
		roiManager("Show All");
		run("Enhance Contrast...", "saturated=0.3");
		roiManager("Measure");
		run("Summarize");
		waitForUser("Cell ROIs");
		roi = roiManager("Count");
		run("Clear Results");
	}

	//Saves cell ROI's
	roiManager("save", cellroi);
	roiManager("reset");
```

```
	return roi;
}

//---------------------------------------------------------------------
//		Beginning of script
//---------------------------------------------------------------------

//Set measurements for results window
run("Set Measurements...", "area mean standard");
//Sets options for "Make Binary" function
run("Options...", "iterations=2 count=1 black do=Nothing");

//User selects image analysis folder
dir = getDirectory("Choose a folder with images for analysis");
filelist = getFileList(dir);

//Sets slash direction for saving files
if(matches(dir,".*/.*")) {slash = "/";}
else			{slash = "\\";}

//Temp ROI file paths
aggroi = dir+slash+"aggroi.zip";
dapiroi = dir+slash+"nucroi.zip";
cellroi = dir+slash+"cellroi.zip";

//Global variables
rowset = 0;
rowagg = 0;
```

```
rowcell = 0;

lower = 15000;

upper = 65535;

dapislice=newArray(1,1,1);

aggslice=newArray(2,1,1);

cellslice=newArray(3,1,1);


//----------------------------------------------------------------------

//                          SETUP

//----------------------------------------------------------------------

if(getBoolean("Choose settings for analysis","Setup","Manual input"))

{

        //Define channel, slice, frame for nuclei, aggregates and cells

        suffixmatch = false;

        for(j=0;j<lengthOf(filelist);j++)

        {

                //Check for compatible file types

                filename = filelist[j];

                if(indexOf(filename,".")>0)

                {

                        filenamesuffix = substring(filename, lastIndexOf(filename, ".")+1,
lengthOf(filename));

                        for(k=0;k<lengthOf(suffixes);k++)

                        {

                                if(matches(filenamesuffix,suffixes[k]))

                                {

                                        suffixmatch = true;

                                        k = lengthOf(suffixes);

                                        j = lengthOf(filelist);
```

```
				}
			}
		}
	}
	if(suffixmatch==false) {exit("No images found in\n"+dir);}


	//Open first image
	bioformsettings = "open=[" + dir + filename + "] autoscale color_mode=Default rois_import=[ROI manager] view=Hyperstack stack_order=XYCZT";
	run("Bio-Formats", bioformsettings);


	waitForUser("Select nuclei channel\n(and/or slice & frame)");
	Stack.getPosition(dapislice[0],dapislice[1],dapislice[2]);
	waitForUser("Select aggregate channel\n(and/or slice & frame)");
	Stack.getPosition(aggslice[0],aggslice[1],aggslice[2]);
	waitForUser("Select cell body channel\n(and/or slice & frame)\nIf no cell body stain,\nselect nuclei channel");
	Stack.getPosition(cellslice[0],cellslice[1],cellslice[2]);
	close();


	//Iterate through images to set threshold limits
	while(getBoolean("Current aggregate threshold:\nupper - "+lower+" lower - "+upper+"\nGet image and adjust threshold?","Get image","Next step"))
	{


		tempfilepath = File.openDialog("Image for aggregate thresholding");
		bioformsettings = "open=[" + tempfilepath + "] autoscale color_mode=Default rois_import=[ROI manager] view=Hyperstack stack_order=XYCZT";
		run("Bio-Formats", bioformsettings);
```

```
		Stack.setPosition(aggslice[0],aggslice[1],aggslice[2]);
		aggnum = AggregateROI(getTitle(),dapiroi,aggroi,true,lower,upper,0,1000000,false);
		getThreshold(lower,upper);
		close("Threshold");
		close("*");
	}
	//Iterate through images to display aggregate sizes and distances from nucleus
	while(getBoolean("Determine aggregate max & min size cutoff and perinuclear zone
cutoff\nGet image to measure and display aggregate size\nand distance from nucleus","Get
image","Next step"))
	{
		//Get image for setup
		tempfilepath = File.openDialog("Image for aggregate size and distance");
		bioformsettings = "open=[" + tempfilepath + "] autoscale color_mode=Default
rois_import=[ROI manager] view=Hyperstack stack_order=XYCZT";
		run("Bio-Formats", bioformsettings);

		//Find nuclei ROIs
		Stack.setPosition(dapislice[0],dapislice[1],dapislice[2]);
		run("Duplicate...","title=template1");
		nucarea = NucleiROI("template1",dapiroi,50,5,true);
		close("template1");

		//Find aggregate ROIs
		Stack.setPosition(aggslice[0],aggslice[1],aggslice[2]);
		run("Duplicate...","title=template1");
		aggnum = AggregateROI("template1",dapiroi,aggroi,false,lower,upper,0,1000000,true);
		close("template1");

		roiManager("open",aggroi);
```

```
roiManager("open",dapiroi);


//Determine distance from closest nuclei
//NOTE Distance calculation is a shortcut used for the setup process, during actual
aggregate
//quantification each aggregate will be assigned a cell and its respective nucleus
for(j=0;j<aggnum;j++)
{
	mindis = 10000;
	roiManager("Select",j);
	getSelectionCoordinates(x,y);
	Array.getStatistics(x, min, max, xc, stdDev);
	Array.getStatistics(y, min, max, yc, stdDev);

	for(k=aggnum;k<roiManager("Count");k++)
	{
		roiManager("Select",k);
		getSelectionCoordinates(X,Y);
		Array.getStatistics(X, min, max, Xc, stdDev);
		Array.getStatistics(Y, min, max, Yc, stdDev);

		if(sqrt(pow(xc-Xc,2) + pow(yc-Yc,2))<500)
		{
			for(l=0;l<X.length;l++)
			{
				for(m=0;m<x.length;m++)
				{
					dis = sqrt(pow(x[m]-X[l],2) + pow(y[m]-Y[l],2));
```

```
                                        if(dis<mindis){mindis=dis;}
                                    }
                                }
                            }
                        }
                        roiManager("Select",j);
                        getStatistics(area);
                        roiManager("Rename","dis "+round(mindis)+" size "+area);
                    }

                    //Display summary stats in the results window
                roiManager("select",Array.slice(Array.getSequence(roiManager("Count")),aggnum,roiManager("
Count")));
                    roiManager("Delete");
                    roiManager("deselect");
                    roiManager("Measure")
                    run("Summarize");

                    //Create image with labels overlayed
                    run("Make Composite");
                    run("From ROI Manager");
                    run("Labels...", "color=white font=10 show use draw");
                    waitForUser("Write down perinuclear distance and\naggregate size cutoff to input
later\nUse '+' and '-' keys to zoom");
                    roiManager("reset");
                    run("Clear Results");
                    close("*");
                }
```

```
//Present user with strictness options for segementation and thresholding cell bodies

while(getBoolean("Optimize cell body processing\nGet image to show three levels of processing: 2.5, 5 & 7.5\nfor both segmentation and thresholding methods","Get image","Next step (settings)"))

{

    tempfilepath = File.openDialog("Image for cell body procesing");

    bioformsettings = "open=[" + tempfilepath + "] autoscale color_mode=Default rois_import=[ROI manager] view=Hyperstack stack_order=XYCZT";

    run("Bio-Formats", bioformsettings);

    id=getImageID();


    Stack.setPosition(dapislice[0],dapislice[1],dapislice[2]);

    run("Duplicate...","title=Dapi duplicate channels="+dapislice[0]+" slices="+dapislice[1]);

    nucarea = NucleiROI("Dapi",dapiroi,50,5,true);

    selectImage(id);

    Stack.setPosition(cellslice[0],cellslice[1],cellslice[2]);

    run("Duplicate...","title=Strictness duplicate channels="+cellslice[0]+" slices="+cellslice[1]);

    for(i=2.5;i<10;i=i+2.5)

    {

        //Segmentation

        run("Merge Channels...", "c1=Dapi c2=Strictness create keep");

        run("Stack to RGB");

        run("16-bit");

        run("Enhance Local Contrast (CLAHE)", "blocksize=249 histogram=240 maximum=18 mask=*None* fast_(less_accurate)");

        run("Subtract Background...", "rolling=300");

        run("Gaussian Blur...", "sigma=10");

        run("Find Maxima...", "prominence="+(i*4)+" strict exclude output=[Segmented Particles]");
```

```
run("Analyze Particles...", "size=0-inifinty pixel circularity=0.00-1.00 add");
close("Composite*");
selectWindow("Strictness");
run("Duplicate...","title=Segmentation_"+i+"_strictness");
run("From ROI Manager");
run("Overlay Options...", "stroke=none width=0 fill=none");
roiManager("Reset");


//Thresholding
selectWindow("Strictness");
run("Duplicate...","title=temp");
run("Enhance Contrast...", "saturated=10 normalize");
getStatistics(area, mean);
run("Subtract...","value="+(mean*(i/5)));
run("Median...", "radius=20");
run("Make Binary");
roiManager("Open",dapiroi);
roiManager("Select",Array.getSequence(roiManager("Count")));
Roi.setFillColor("white");
roiManager("Fill");
roiManager("Reset");
run("Make Binary");
run("Dilate");
run("Analyze Particles...", "size=0-inifinty pixel circularity=0.00-1.00 add");
close("temp");
selectWindow("Strictness");
run("Duplicate...","title=Thresholding_"+i+"_strictness");
if(roiManager("Count")>0)
```

```
                {
                        run("From ROI Manager");
                        run("Overlay Options...", "stroke=none width=0 fill=none");
                        roiManager("Reset");
                }
        }
        selectImage(id);
        close();
        close("Dapi");
        run("Tile");
        waitForUser("Write down ideal strictness level and method from image title\nAny value
between 1-10 may be used");
        close("*");
    }
    run("Clear Results");
}

//Create the main settings window
Dialog.create("AggreCount settings");
Dialog.addHelp("https://www.biorxiv.org/content/10.1101/2020.07.25.221267v1");
Dialog.setInsets(-10, 120, 3);
Dialog.addMessage("                              _____                    __   \n"+
"    /\\                        /  ___|                  |  | \n"+
"   /  \\  _ _ _ _ _ __ _|   |      __ _ _ __  |   |_ \n"+
"  / /\\ \\/ _  |/ _  |  __/ _ \\  |     / _ \\ | |  |  |  _ \\|  __|\n"+
" / /_\\ \\ (_| | (_| | | |  __/   |__| (_)|  |_| | | |   |   |_ \n"+
" /_/   \\_\\ _, |\\_, |_|  \\__|\\_____\\__/ \\_,_|_| |_|\\__|\n"+
"         _/  | _/  |                              \n"+
"        |__/ |__/");
```

```
Dialog.addMessage("Aggregate thresholding:\n");
Dialog.addNumber("Lower: ",lower);
Dialog.addToSameRow();
Dialog.addNumber("Upper: ",upper);
Dialog.addMessage("Perinuclear distance cutoff\n");
Dialog.addNumber("Distance: ", 10, 0, 4, "pixels");
Dialog.addNumber("Aggresome min size:", 5, 1, 4, "um^2");
Dialog.addNumber("Aggregate min size:", 0.1, 2, 4, "um^2");
Dialog.addToSameRow();
Dialog.addNumber("Max size:", 25, 0, 2, "um^2");
Dialog.addNumber("Nuclei size:", 50, 0, 4, "um^2");
Dialog.addToSameRow();
Dialog.addNumber("Strictness:", 5, 0, 2, "(1-10)");
Dialog.addNumber("Cell size", 75, 0, 4, "um^2");
Dialog.addToSameRow();
Dialog.addNumber("Strictness:", 5, 0, 2, "(1-10)");
Dialog.addNumber("Nuclei: ",dapislice[0], 0, 2, "channel");
Dialog.addToSameRow();
Dialog.addNumber("",dapislice[1], 0, 2, "slice");
Dialog.addToSameRow();
Dialog.addNumber("",dapislice[2], 0, 2, "frame");
Dialog.addNumber("Aggregates: ",aggslice[0], 0, 2, "channel");
Dialog.addToSameRow();
Dialog.addNumber("",aggslice[1], 0, 2, "slice");
Dialog.addToSameRow();
Dialog.addNumber("",aggslice[2], 0, 2, "frame");
Dialog.addNumber("Cell bodies: ",cellslice[0], 0, 2, "channel");
Dialog.addToSameRow();
Dialog.addNumber("",cellslice[1], 0, 2, "slice");
```

```
Dialog.addToSameRow();
Dialog.addNumber("",cellslice[2], 0, 2, "frame");
Dialog.addCheckbox("Find cell bodies?", 1)
Dialog.addChoice("Cell processing  method", newArray("Segmentation","Thresholding"));
Dialog.addCheckbox("Save results?",1);
Dialog.addToSameRow();
Dialog.addCheckbox("Batch mode?",0);
Dialog.show();
lower = Dialog.getNumber();
upper = Dialog.getNumber();
perinucdis = Dialog.getNumber();
aggresomecut = Dialog.getNumber();
aggcutmin = Dialog.getNumber();
aggcutmax = Dialog.getNumber();
nuccut = Dialog.getNumber();
nucstrictness = Dialog.getNumber();
cellcut = Dialog.getNumber();
strictness = Dialog.getNumber();
dapislice[0] = Dialog.getNumber();
dapislice[1] = Dialog.getNumber();
dapislice[2] = Dialog.getNumber();
aggslice[0] = Dialog.getNumber();
aggslice[1] = Dialog.getNumber();
aggslice[2] = Dialog.getNumber();
cellslice[0] = Dialog.getNumber();
cellslice[1] = Dialog.getNumber();
cellslice[2] = Dialog.getNumber();
cellfind = Dialog.getCheckbox();
segmentation = Dialog.getChoice();
```

```
saveR = Dialog.getCheckbox();
batch = Dialog.getCheckbox();

//Open dataset tables and close previously open tables
if(isOpen("Dataset_summary")) {close("Dataset_summary");}
Table.create("Dataset_summary");
if(isOpen("Dataset_cells")) {close("Dataset_cells");}
if(cellfind) {Table.create("Dataset_cells");}
if(isOpen("Dataset_aggregates")) {close("Dataset_aggregates");}
Table.create("Dataset_aggregates");
if(isOpen("Data")) {close("Data");}

if(matches(segmentation,"Segmentation")) {segmentation=true;}

//Creates save folder and saves aggrecount settings if Save results is checked
if(saveR)
{
	i=0;
	do
	{
		i=i+1;
		newdir = dir+"AC_analysis"+i;
	} while(File.exists(newdir));

	File.makeDirectory(newdir);

	setvar = newArray("Lower","Upper","perinuclear distance","aggresome size","minimum aggregate size","maximum aggregate size","minimum nuclei size","nuclei strictness","minimum cell size","cell strictness","nuclei channel","nuclei stack","nuclei frame","aggregate channel","aggregate
```

```
stack","aggregate frame","cell channel","cell stack","cell frame","Find cells?","Segmentation?","Batch mode");

	setval = newArray(lower,upper,perinucdis,aggresomecut,aggcutmin,aggcutmax,nuccut,nucstrictness,cellcut,strictness,dapislice[0],dapislice[1],dapislice[2],aggslice[0],aggslice[1],aggslice[2],cellslice[0],cellslice[1],cellslice[2],cellfind,segmentation,batch);


	Table.create("Settings");
	for(i=0;i<lengthOf(setval);i++)
	{
	Table.set("Variable",i,setvar[i]);
	Table.set("Value",i,setval[i]);
	}
	Table.save(newdir+slash+"aggresettings.txt");
	close("Settings");
}


ubq = "Aggregates";
dapi = "Nuclei";
cell = "Cells";


if(isOpen(ubq)) {close(ubq);}
if(isOpen(dapi)) {close(dapi);}
if(isOpen(cell)) {close(cell);}


//---------------------------------------------------------------------------
//		Image analysis section
//---------------------------------------------------------------------------


for(i=0;i<lengthOf(filelist);i++) {  //Cycle through each file in directory
```

```
filename = filelist[i];

//Ask user to analyze images if not batch mode
if(batch) {cont = true;}
else      {cont = getBoolean("Next file:\n"+filename+"\nContinue?","Continue","Skip");}

//Check for compatible file types
suffixmatch = false;
if(indexOf(filename,".")>0)
{
        filenamesuffix = substring(filename, lastIndexOf(filename, ".")+1, lengthOf(filename));
        for(j=0;j<lengthOf(suffixes);j++)
        {
                if(matches(filenamesuffix,suffixes[j]))
                {
                        suffixmatch = true;
                        j = lengthOf(suffixes);
                }
        }
}

if(suffixmatch && cont) { //Only accept files that are compatible with Bio-Formats importer

if(saveR) {savepath = newdir+slash+substring(filename,0,lengthOf(filename)-4);} //Create save path in the AC_analysis folder created earlier

//Maintain batch mode for ROI manager
close("ROI Manager");
```

```
setBatchMode(batch);

roiManager("reset");


//Open next image

bioformsettings = "open=[" + dir + filename + "] autoscale color_mode=Default rois_import=[ROI manager] view=Hyperstack stack_order=XYCZT";

run("Bio-Formats", bioformsettings);

id = getImageID();


//opens each channel as a separate image

selectImage(id);

Stack.setPosition(dapislice[0],dapislice[1],dapislice[2]);

run("Duplicate...","title="+dapi+" duplicate channels="+dapislice[0]+" slices="+dapislice[1]);

selectImage(id);

Stack.setPosition(aggslice[0],aggslice[1],aggslice[2]);

run("Duplicate...","title="+ubq+" duplicate channels="+aggslice[0]+" slices="+aggslice[1]);

selectImage(id);

if(cellfind)

{

	Stack.setPosition(cellslice[0],cellslice[1],cellslice[2]);

	run("Duplicate...","title="+cell+" duplicate channels="+cellslice[0]+" slices="+cellslice[1]);

}

selectImage(id);

close();


nucarea = NucleiROI(dapi,dapiroi,nuccut,nucstrictness,batch); //Determine nuclei ROI (see function above)


aggnum = AggregateROI(ubq,dapiroi,aggroi,false,lower,upper,aggcutmin,aggcutmax,batch); //Determine aggregate ROI (see function above)
```

```
//Create aggregate data arrays
aggID = newArray(aggnum);
aggArea = newArray(aggnum);
aggMean = newArray(aggnum);
aggSTD = newArray(aggnum);
aggLoc = newArray(aggnum);
aggdis = newArray(aggnum+1);
aggClass = newArray(aggnum);
aggCell = newArray(aggnum);

//If user selects "Find cells" setting
if(cellfind)
{
	cellnum = cellROI(cell,dapiroi,aggroi,cellroi,cellcut,nucarea,batch,segmentation,strictness);
//Finds cell ROIs (see function above)

	roiManager("Open",cellroi);

	//Adds cell ROI data to the data table and labels cells
	cellArea = newArray(cellnum);
	cellMean = newArray(cellnum);
	cellSTD = newArray(cellnum);
	for(j=0;j<cellnum;j++)
	{
		roiManager("select",j);
		getStatistics(cellArea[j], cellMean[j], min, max, cellSTD[j]);
	}
	roiManager("reset");
```

```
//Beginning of sequence that assigns nuclei and aggregates to cells
roiManager("open", cellroi);
roiManager("open", dapiroi);
nuccellnum = roiManager("Count");
if(aggnum>0) {roiManager("open", aggroi);}

//Initialize dataset_cells arrays
cellnuccount = newArray(cellnum);
cellaggcount = newArray(cellnum);
cellaggarea = newArray(cellnum);
cellcytagg = newArray(cellnum);
cellcytaggarea = newArray(cellnum);
cellpnagg = newArray(cellnum);
cellpnaggarea = newArray(cellnum);
cellnucagg = newArray(cellnum);
cellnucaggarea = newArray(cellnum);
cellaggresomecount = newArray(cellnum);
cellaggresomearea = newArray(cellnum);
cellpnagglargest = newArray(cellnum);

//dataset_summary variables
cellcount=0;
totagg=0;
totaggarea=0;
totcytagg=0;
totcytaggarea=0;
totpnagg=0;
totpnaggarea=0;
```

```
totnucagg=0;

totnucaggarea=0;

totaggresome=0;

percentagg=0;

percentaggresome=0;

aggrow=0;


for(j=0;j<cellnum;j++) //For each cell

{

    cellaggnum = newArray(aggnum);

    o=0;

    cellnucroi = newArray(nuccellnum);

    p=0;


    for(k=cellnum;k<roiManager("Count");k++) //For each ROI that's NOT a cell

    {

        roiManager("Select",newArray(j,k));

        roiManager("AND");

        if(selectionType>-1) //If nuclei or aggregate is contained in the cell

        {

            getStatistics(area);

            aggIDdel = Array.deleteValue(aggID,k);

            if (k<roiManager("count")-aggnum) //If ROI is a nucleus

            {

                if(area>nucarea){cellnuccount[j] = cellnuccount[j] +
floor(area/nucarea);} //If the area is LARGER than an average nucleus, count it as multiple based on size

                else        {cellnuccount[j] = cellnuccount[j] + 1;}

                cellnucroi[p] = k;

                p = p + 1;
```

```
}
else  if (lengthOf(aggID) == lengthOf(aggIDdel))
{
	aggID[aggrow] = k;
	cellnucroi = Array.slice(cellnucroi,0,p); //Create array of all nuclei ROIs within cell ROI
	nuclear = false;
	aggdis[aggrow] = 10000;
	roiManager("Select",k);
	getStatistics(areak);
	getSelectionCoordinates(x,y);

	for(n=0;n<lengthOf(cellnucroi);n++) //Cycle through each nucleus ROI
	{
		roiManager("Select",cellnucroi[n]);
		getSelectionCoordinates(X,Y);

		roiManager("Select",newArray(cellnucroi[n],k));
		roiManager("AND");
		getStatistics(area);

		if(area==areak) //If the aggregate is completely contained within the nuclei
		{
			nuclear = true;
			n = lengthOf(cellnucroi);
		}
```

```
for(l=0;l<X.length;l++) //Nested for loops calc the distance between each point of the agg and nuclei
{
    for(m=0;m<x.length;m++)
    {
        dis = sqrt(pow(x[m]-X[l],2) + pow(y[m]-Y[l],2));
        if(dis<aggdis[aggrow])
            {aggdis[aggrow]=dis;}
        if(dis<2) {n = lengthOf(cellnucroi);}
    }
}
}
//adds aggregate and cell data to respective arrays
roiManager("select",k);
getStatistics(aggArea[aggrow], aggMean[aggrow], min, max, aggSTD[aggrow]);

if(aggdis[aggrow]<=perinucdis && aggArea[aggrow]>=aggresomecut)
{
    aggClass[aggrow] = "aggresome";
    cellaggresomecount[j] = cellaggresomecount[j] + 1;
    cellaggresomearea[j] = cellaggresomearea[j] + aggArea[aggrow];
}
else
{
    aggClass[aggrow] = "aggregate";
}
```

```
if(nuclear && aggdis[aggrow]>perinucdis)
{
	aggLoc[aggrow] = "nuclear";
	cellnucagg[j] = cellnucagg[j] + 1;
	cellnucaggarea[j] = cellnucaggarea[j] +
aggArea[aggrow];
}
else if(aggdis[aggrow]<=perinucdis)
{
	aggLoc[aggrow] = "perinuclear";
	cellpnagg[j] = cellpnagg[j] + 1;
	cellpnaggarea[j] = cellpnaggarea[j] + aggArea[aggrow];

	if(cellpnagglargest[j]<aggArea[aggrow])
{cellpnagglargest[j]=aggArea[aggrow];}
}
else
{
	aggLoc[aggrow] = "cytosolic";
	cellcytagg[j] = cellcytagg[j] + 1;
	cellcytaggarea[j] = cellcytaggarea[j] + aggArea[aggrow];
}

cellaggcount[j] = cellaggcount[j] + 1;
cellaggarea[j] = cellaggarea[j] + aggArea[aggrow];

//Assign aggregate to that cell
aggCell[aggrow] = j+1;
```

```
                    aggrow = aggrow + 1;
                }
            }
        }

        //Update image variables with new counts
        totagg = totagg + cellaggcount[j];
        totaggarea= totaggarea + cellaggarea[j];
        totaggresome = totaggresome + cellaggresomecount[j];
        totcytagg = totcytagg + cellcytagg[j];
        totcytaggarea = totcytaggarea + cellcytaggarea[j];
        totnucagg = totnucagg + cellnucagg[j];
        totnucaggarea = totnucaggarea + cellnucaggarea[j];
        totpnagg = totpnagg + cellpnagg[j];
        totpnaggarea = totpnaggarea + cellpnaggarea[j];
        if(cellaggcount[j]>0 && cellaggcount[j]<cellnuccount[j]){percentagg = percentagg + cellaggcount[j];}
        else if(cellaggcount[j]>0 && cellaggcount[j]>=cellnuccount[j]){percentagg = percentagg + cellnuccount[j];}
        if(cellaggresomecount[j]>0 && cellaggresomecount[j]<cellnuccount[j]){percentaggresome = percentaggresome + cellaggresomecount[j];}
        else if(cellaggresomecount[j]>0 && cellaggresomecount[j]>=cellnuccount[j]){percentaggresome = percentaggresome + cellnuccount[j];}
        cellcount = cellcount + cellnuccount[j];
    }

    //After cycling through all cells, add image summary data to dataset table
    selectWindow("Dataset_summary");
    Table.set("File",rowset,filename);
```

```
Table.set("Cells",rowset,cellcount);
Table.set("Aggregates per cell",rowset,(totagg/cellcount));
Table.set("Aggregate area per cell",rowset,(totaggarea/cellcount));
Table.set("Avg aggregate size",rowset,(totaggarea/totagg));
Table.set("Cells with aggregates",rowset,((percentagg)));
Table.set("% cells with aggregates",rowset,(percentagg/cellcount));
Table.set("Cells with aggresome",rowset,((percentaggresome)));
Table.set("% cells with aggresome",rowset,(percentaggresome/cellcount));
Table.set("% aggs perinuclear",rowset,(totpnagg/totagg));
Table.set("% aggs cytosolic",rowset,(totcytagg/totagg));
Table.set("% aggs nuclear",rowset,(totnucagg/totagg));
Table.set("Perinuclear aggregates per cell",rowset,(totpnagg/cellcount));
Table.set("Perinuclear agg area per cell",rowset,(totpnaggarea/cellcount));
Table.set("Cytosolic aggregates per cell",rowset,(totcytagg/cellcount));
Table.set("Cytosolic agg area per cell",rowset,(totcytaggarea/cellcount));
Table.set("Nuclear aggregate per cell",rowset,(totnucagg/cellcount));
Table.set("Nuclear agg area per cell",rowset,(totnucaggarea/cellcount));

Table.update;

//print data to cells window
selectWindow("Dataset_cells");
for(j=0;j<cellnum;j++)
{
	Table.set("File", rowcell, filename);
	Table.set("Nuclei", rowcell, cellnuccount[j]);
	Table.set("Aggregates", rowcell, cellaggcount[j]);
	Table.set("Aggregate area", rowcell, cellaggarea[j]);
	Table.set("Avg aggregate size", rowcell, cellaggarea[j]/cellaggcount[j]);
```

```
		Table.set("Cytosolic aggregates", rowcell, cellcytagg[j]);
		Table.set("Cytosolic agg area", rowcell, cellcytaggarea[j]);
		Table.set("Avg cyt agg size", rowcell, cellcytaggarea[j]/cellcytagg[j]);
		Table.set("Perinuclear aggregates", rowcell, cellpnagg[j]);
		Table.set("Perinuclear agg area", rowcell, cellpnaggarea[j]);
		Table.set("Avg peri agg size", rowcell, cellpnaggarea[j]/cellpnagg[j]);
		Table.set("Nuclear aggregates", rowcell, cellnucagg[j]);
		Table.set("Nuclear agg area", rowcell, cellnucaggarea[j]);
		Table.set("Avg nuc agg size", rowcell, cellnucaggarea[j]/cellnucagg[j]);
		Table.set("Largest perinuclear agg", rowcell, cellpnagglargest[j]);
		Table.set("Area", rowcell, cellArea[j]);
		Table.set("Mean", rowcell, cellMean[j]);
		Table.set("StdDev", rowcell, cellSTD[j]);
		rowcell=rowcell+1;
	}
	Table.update;

	rowset = rowset+1; //Proceed to next row in dataset table for next image
}
else //If not aggregate ROIs are present
{

	//open aggregate and nuclei ROI to determine distances
	if(aggnum>0) {roiManager("open",aggroi);}
	roiManager("open",dapiroi);
	selectWindow(ubq);

	//Cycle through each aggregate to determine distance from nearest nuclei
	for(j=0;j<aggnum;j++)
```

```
{
	nuclear = false;
	aggdis[j] = 10000;
	mindisC = 10000;
	centroiddis = 500;

	roiManager("Select",j);
	getStatistics(areaj);
	getSelectionCoordinates(x,y);
	Array.getStatistics(x, min, max, xc); //Get x centroid of aggregate
	Array.getStatistics(y, min, max, yc); //get y centroid of aggregate

	for(k=aggnum;k<roiManager("Count");k++) //Cycle through each nucleus ROI
	{
		roiManager("Select",k);
		getSelectionCoordinates(X,Y);
		Array.getStatistics(X, min, max, Xc, stdDev); //get x centroid of nucleus
		Array.getStatistics(Y, min, max, Yc, stdDev); //get y centroid of nucleus
		disC = sqrt(pow(xc-Xc,2) + pow(yc-Yc,2));
		if(disC<mindisC) {mindisC=disC;disCi=k;}

		if(disC<centroiddis) //Only calculate distances for agg + nuclei with centroids 
within 500 pixels
		{
			roiManager("Select",newArray(j,k));
			roiManager("AND");
			getStatistics(area);
```

```
					if(area==areaj) //If the aggregate is completely contained within the
nuclei
					{
						nuclear = true;
						k = roiManager("Count");
					}

					for(l=0;l<X.length;l++) //Nested 'for' loops calc the distance between
each point of the agg and nuclei
					{
						for(m=0;m<x.length;m++)
						{
							dis = sqrt(pow(x[m]-X[l],2) + pow(y[m]-Y[l],2));
							if(dis<aggdis[j])
							{aggdis[j]=dis;}
							if(dis==0)
							{k = roiManager("Count");}
						}
					}
				}
				if(k==roiManager("Count") && aggdis[j]==10000) {k=disCi-
1;centroiddis=mindisC+1;}
			}
			//adds aggregate data to the arrays
			roiManager("select",j);
			getStatistics(aggArea[j], aggMean[j], min, max, aggSTD[j]);
			if(aggdis[j]<=perinucdis && aggArea[j]>=aggresomecut) {aggClass[j] = "aggresome";}
			else					{aggClass[j] = "aggregate";}
			if(nuclear && aggdis[j]>perinucdis)		{aggLoc[j] = "nuclear";}
			else if(aggdis[j]<=perinucdis)		{aggLoc[j] = "perinuclear";}
```

```
	else					{aggLoc[j] = "cytosolic";}
}
roiManager("reset");


//Extracts cell count via nuclei number
roiManager("open", dapiroi);
cellcount = 0;
for(j=0;j<roiManager("Count");j++)
{
	roiManager("select",j);
	getStatistics(area);
	tempcount = round(area/nucarea);
	if(tempcount>0) {cellcount = cellcount + tempcount;}
	else {cellcount = cellcount + 1;}
}

//Dataset_summary variables
totagg=0;
totaggarea=0;
totcytagg=0;
totcytaggarea=0;
totpnagg=0;
totpnaggarea=0;
totnucagg=0;
totnucaggarea=0;
totaggresome=0;
totaggresomearea=0;
for(j=0;j<lengthOf(aggArea);j++) //For each aggregate
```

```
{
	if(matches(aggClass[j],"aggresome"))
	{
		totaggresome = totaggresome + 1;
		totaggresomearea = totaggresomearea + area;
	}

	if(matches(aggLoc[j],"cytosolic"))
	{
		totcytagg = totcytagg + 1;
		totcytaggarea = totcytaggarea + area;
	}
	else if(matches(aggLoc[j],"perinuclear"))
	{
		totpnagg = totpnagg + 1;
		totpnaggarea = totpnaggarea + area;
	}
	else
	{
		totnucagg = totnucagg + 1;
		totnucaggarea = totnucaggarea + area;
	}

	totagg = totagg + 1;
	totaggarea = totaggarea + area;
}

//print data to summary window
selectWindow("Dataset_summary");
```

```
		Table.set("File",rowset,filename);
		Table.set("Cells",rowset,cellcount);
		Table.set("Aggregates per cell",rowset,(totagg/cellcount));
		Table.set("Aggregate area per cell",rowset,(totaggarea/cellcount));
		Table.set("Average aggregate size",rowset,(totaggarea/totagg));
		Table.set("Total aggresomes",rowset,(totaggresome));
		Table.set("% aggs perinuclear",rowset,(totpnagg/totagg));
		Table.set("% aggs cytosolic",rowset,(totcytagg/totagg));
		Table.set("% aggs nuclear",rowset,(totnucagg/totagg));
		Table.set("Perinuclear aggregates per cell",rowset,(totpnagg/cellcount));
		Table.set("Perinuclear agg area per cell",rowset,(totpnaggarea/cellcount));
		Table.set("Cytosolic aggregates per cell",rowset,(totcytagg/cellcount));
		Table.set("Cytosolic agg area per cell",rowset,(totcytaggarea/cellcount));
		Table.set("Nuclear aggregate per cell",rowset,(totnucagg/cellcount));
		Table.set("Nuclear agg area per cell",rowset,(totnucaggarea/cellcount));

		rowset = rowset+1;
		Table.update;

		roiManager("open", aggroi);
	}

	//print data to aggregate window
	selectWindow("Dataset_aggregates");
	for(j=0;j<lengthOf(aggArea);j++)
	{
		if(aggArea[j]>0)
		{
			Table.set("File", rowagg, filename);
```

```
			Table.set("Location", rowagg, aggLoc[j]);
			Table.set("Distance",rowagg, aggdis[j]);
			Table.set("Area", rowagg, aggArea[j]);
			Table.set("Mean", rowagg, aggMean[j]);
			Table.set("StdDev",rowagg, aggSTD[j]);
			rowagg=rowagg+1;
		}
}
Table.update;

row=0;
//print data for the specific image file
Table.create("Data");
if(cellfind) //print cell data to image data file
{
		for(j=0;j<lengthOf(cellArea);j++)
		{
			Table.set("Class",row,"Cell");
			Table.set("Cell #",row,j+1);
			Table.set("Location",row,"NA");
			Table.set("Area",row,cellArea[j]);
			Table.set("Mean",row,cellMean[j]);
			Table.set("Stdev",row,cellSTD[j]);
			Table.set("Nuclei",row,cellnuccount[j]);
			Table.set("Aggregates",row,cellaggcount[j]);
			Table.set("Aggregate area",row,cellaggarea[j]);
			Table.set("Cytosolic agg",row,cellcytagg[j]);
			Table.set("Cytosolic agg area",row,cellcytaggarea[j]);
			Table.set("Perinuclear agg",row,cellpnagg[j]);
```

```
			Table.set("Perinuclear agg area",row,cellpnaggarea[j]);
			Table.set("Nuclear agg",row,cellnucagg[j]);
			Table.set("Nuclear agg area",row,cellnucaggarea[j]);
			Table.set("Aggresomes",row,cellaggresomecount[j]);
			Table.set("Aggresome area",row,cellaggresomearea[j]);
			row=row+1;
		}
}

for(j=0;j<lengthOf(aggArea);j++) //print aggregate data to image data field
{
		if(aggArea[j]>0)
		{
			Table.set("Class",row,aggClass[j]);
			Table.set("Cell #",row,aggCell[j]);
			Table.set("Location",row,aggLoc[j]);
			Table.set("Area",row,aggArea[j]);
			Table.set("Mean",row,aggMean[j]);
			Table.set("Stdev",row,aggSTD[j]);
			Table.set("Distance",row,aggdis[j]);
			row=row+1;
		}
}

if(batch==false)
{
		roiManager("show all without labels")
		Table.update;
		waitForUser("Click 'OK' for next image");
```

```
}
roiManager("reset");

//save function for image data and ROIs
if(saveR)
{
	selectWindow("Data");
	save(savepath+"_analysis.txt");
	close(substring(filename,0,lengthOf(filename)-4)+"_analysis.txt");
	roisave = savepath + "_rois.zip";
	roiManager("Reset");
	if(cellfind){roiManager("Open",cellroi);}
	roiManager("Open",dapiroi);
	if(aggnum>0){roiManager("Open",aggroi);}
	roiManager("save",roisave);
	roiManager("Reset");
}
close("*");

//delete temporary roi files
d=File.delete(aggroi);
d=File.delete(dapiroi);
d=File.delete(cellroi);
}
}
//save dataset windows
if(saveR)
{
	selectWindow("Dataset_summary");
```

```
	save(newdir+slash+"dataset_summary.txt");


	selectWindow("Dataset_aggregates");
	save(newdir+slash+"dataset_aggregates.txt");


	if(cellfind)
	{
		selectWindow("Dataset_cells");
		save(newdir+slash+"dataset_cells.txt");
	}


	f = File.open(newdir+slash+"dataset_description.txt");
	print(f,"Analysis output\nAll data files are saved as tab-delimited .txt files.\nThese may be easily imported into excel or another data processing software.\nIn addition to the data tables described below, ROIs for each image are saved as\n.zip files within the AC_analysis folder. Each ROI zip file contains ROIs for cells, nuclei, and aggregates\nin that order.\n\nDataset_summary\nSummary data by image\n	File – Filename\n	Cells – Number of cells imaged (uses # of nuclei)\n	Aggregates per cell – Total aggregates in image divided by total number of cells (nuclei)\n	Aggregate area per cell – Total aggregate area in image divided by total number of cells\n	Avg aggregate size – Total aggregate area divided by total aggregates\n	Cells with aggregates* – Total number of cells with any type of aggregate\n	% cells with aggregates* – Percentage of all cells with any type of aggregate\n	Cells with aggresome* – Total number of cells with an aggregate above the user defined size cutoff within the perinuclear zone (aggresome)\n	% cells with aggresome* – Percentage of all cells with an aggregate above the user defined size cutoff within the perinuclear zone (aggresome)\nTotal aggresomes** - Total number of aggresomes in the image\n	% aggs perinuclear – Perinuclear aggregates divided by total aggregates\n	% aggs cytosolic – Cytosolic aggregates divided by total aggregates\n	% aggs nuclear – Nuclear aggregates divided by total aggregates\n	Perinuclear aggregates per cell – Total perinuclear aggregates divided by total cells (nuclei)\n	Perinuclear aggregate area per cell – Total perinuclear aggregate area divided by total cells (nuclei)\n	Cytosolic aggregates per cell – Total cytosolic aggregates divided by total cells (nuclei)\n	Cytosolic aggregate area per cell – Total cytosolic aggregate area divided by total cells (nuclei)\n	Nuclear aggregates per cell – Total nuclear aggregates divided by total cells (nuclei)\n	Nuclear aggregate area per cell – Total nuclear aggregate area divided by total cells (nuclei)\n\nDataset_cells*\nSummary data by cell\n	File – Filename of the image that contains the cell\n	Nuclei – Number of nuclei contained within a cell ROI\n	Aggregates – Total number of aggregates within a cell\n	Aggregate area – Total aggregate area within a cell (um^2)\n	Avg aggregate size – Total aggregates within the cell divided by total aggregate area within the cell\n	Cytosolic aggregates – Total number of cytosolic aggregates within a cell\n	Cytosolic agg
```

```
area – Total cytosolic aggregate area within a cell\n	Avg cyt agg size - Average cytosolic aggregate size\n	Perinuclear aggregates – Total number of perinuclear aggregates within a cell\n	Perinuclear agg area – Total perinuclear aggregate area within a cell\n	Avg peri agg size - Average perinuclear aggregate size\n		Nuclear aggregates – Total number of nuclear aggregates within a cell\n	Nuclear agg area – Total nuclear aggregate area within a cell\n	Avg nuc agg size - Average nuclear aggregate size\n	Largest perinuclear agg - Size of the largest aggregate in the perinuclear zone\n	Area – Total area of a cell ROI\n	Mean – Mean fluorescent intensity from a cell ROI\n	StDev – Standard deviation of fluorescent intensity from a cell ROI\n\nDataset_aggregates\nSummary data by aggregate\n	File – Filename of image that contains the aggregate\n	Location – Cellular compartment of an aggregate (nuclear, perinuclear, cytosolic)\n	Distance – Distance in pixels between the nearest edge of an aggregate to a nucleus ROI\n	Area – Area of the aggregate\n	Mean – Mean fluorescent intensity from aggregate ROI\n	StDev – Standard deviation of the fluorescent intensity from aggregate ROI \n\n‘Image name’_analysis\nSummary data for a specific image. Contains data for both cell and aggregate ROIs\n	Class – Classification of ROI: cell, aggregate, aggresome\n		Cell # - Cell ID that links aggregates to cells\n	Location – Subcellular location of aggregates (cytosolic, perinuclear, nuclear). Cell ROIs will be labeled “NA”\n	Area – Area of a ROI\n	Mean – Mean fluorescence intensity of a ROI\n		StDev – Standard deviation of fluorescence intensity of a ROI\n
	Nuclei* – Number of nuclei contained within a cell ROI\n		Aggregate ROIs will have “0” in this column\n	Aggregates* – Total number of aggregates in a cell\n	Aggregate ROIs will have “0” in this column\n	Aggregate area* – Total sum of aggregate area in a cell\n		Aggregate ROIs will have “0” in this column\n		Cytosolic agg* – Total number of cytosolic aggregates in a cell\n
	Aggregate ROIs will have “0” in this column\n	Cytosolic agg area* – Total sum of cytosolic aggregate area in a cell\n		Aggregate ROIs will have “0” in this column\n	Perinuclear agg* – Total number of perinuclear aggregates in cell\n		Aggregate ROIs will have “0” in this column\n
	Perinuclear agg area* – Total sum of perinuclear aggregate area in a cell\n		Aggregate ROIs will have “0” in this column\n	Nuclear agg* – Total number of nuclear aggregates in a cell\n
	Aggregate ROIs will have “0” in this column\n	Nuclear agg area* – Total sum of nuclear aggregate area in a cell\n		Aggregate ROIs will have “0” in this column\n	Aggresomes* – Total number of aggresomes in a cell\n		Aggregate ROIs will have “0” in this column\n	Aggresome area* – Total aggresome area in a cell\n	Aggregate ROIs will have “0” in this column\n	Distance – Aggregate distance in pixels from a nucleus ROI perimeter\n	Cell ROIs will have “0” in this column\n*designates fields or data tables that are only present in analyses that use cell ROIs\n** designates fields that are only present in analyses that do not use cell ROIs");


}
```

Instructions for AggreCount analysis

Sample images and macro code are available at:

https://tufts.box.com/s/ys3kktb5ujdnilyqu8mnup3mrtiej6b6

Quick start guide

1. Download the Aggrecount macro
    a. Install via the plugin menu "Install…" option
    b. Or drag and drop onto the ImageJ toolbar to open in the macro editor
2. Place all images for analysis into one folder
    a. All images should have the same channels in the same order
3. Run Aggrecount from either the plugin menu or the "Run" button in the macro editor
4. Select the folder with images for analysis
5. Select "Setup"
6. Follow instructions to designate nuclei, aggregate, and cell body channels
    a. If there is no cell body stain, select nuclei channel
7. Select "Get image" to adjust threshold (applied to all images during analysis)
    a. Choose an image and adjust threshold
    b. Continue until satisfied with threshold
        i. Press "Next step"
8. Select "Get image" to view aggregate size and distance from nucleus
    a. Note size of noise vs signal for size cut off
    b. Note distance from nuclear perimeter
        i. This distance designates the perinuclear zone
    c. Press "Next step"
9. Select "Get image" to view different cell body processing methods and strictness
    a. Note method and strictness from image titles
    b. Press "Next step (settings)"
10. Main settings window
    a. Threshold values are auto-populated from setup
    b. Adjust perinuclear distance, aggregate min/max levels as previously noted
    c. Adjust strictness of cell body processing and method as previously noted
    d. Adjust other settings as desired (see below for explanations)
    e. Batch mode
        i. Unchecked
            1. Macro will pause after acquiring ROIs and show user
            2. Useful to ensure fidelity of processing and ROI capture before running a batch mode analysis
        ii. Checked
            1. Macro will proceed to analyze all images in folder in the background
            2. Useful when processing an entire experiment
11. Data files will be saved after all images have been analyzed in the folder selected
    a. Look for "AC_analysis#" folder

AggreCount analysis setup

Users may decide to follow the setup process outlined below or manually input settings by selecting "Setup" or "Manual input" after selecting image analysis folder. It is recommended that users utilize the set-up process for first time analyses of an experiment to help select proper settings.

1. Open FIJI (v1.52p or later)
2. Download the AggreCount macro
    a. Install using the "Install…" option under the "Plugin" menu in the ImageJ toolbar
    b. Alternatively, drag and drop the Aggrecount macro icon onto the ImageJ toolbar to open Aggrecount in the macro editor
3. Place all images for analysis into one folder
    a. All images supported by the Bio-Formats importer may be used, however, images must contain multiple channels
4. Select the Aggrecount macro from the "Macros" menu under the "Plugins" menu in the ImageJ toolbar
    a. Alternatively, select "Run" from the bottom of the macro editor window
5. Select the folder with images for analysis
6. The macro will automatically open the first image and ask the user to select the channels for nuclei, aggregates, and cell body
    a. Change the channel to the immunostain specified by the window and select "Okay"
    b. If there is no stain for cell bodies, select the nuclei channel
7. The next window will prompt the user to get an image to adjust thresholding
    a. Select "Get image" and choose an image with aggregates
    b. Adjust the threshold so aggregates are highlighted in red with as little background as possible and click "Okay"
        i. Do not close the threshold window
    c. Select "Get image" again and choose an image without aggregates
    d. The previous threshold will be shown, adjust appropriately and click "Okay"
    e. Select "Get image" again and choose an image with aggregates
    f. Adjust threshold as previously mentioned and click okay
    g. Either continue cycling through images to adjust threshold or, when satisfied, select "Next step"
        i. This threshold will be applied to all images in the analysis
8. The next window will prompt the user to get an image for determining aggregate size cutoff and perinuclear distance cutoff
    a. Select "Get image" and choose an image with aggregates
    b. The macro will identify aggregates using the previously set threshold and nuclei using an automated thresholding system
    c. The image will be displayed with the size of each aggregate in square microns as well as the distance from the nucleus perimeter in pixels
    d. Use the "+" and "-" buttons on the keyboard to zoom in and out of the image
    e. Note the smallest and largest size of 'true' aggregates vs noise as well as the distance from the nucleus
        i. The default setting for the perinuclear zone is 10 pixels for 60x magnification images, this may need to be adjusted for images of different magnification
        ii. The default setting for aggregate size is 0.1 – 25mm^2 which is applicable to most aggregate images

    f. Select "Okay" when finished noting sizes and distances
    g. Either continue cycling through images to note sizes and distances or select "Next step" to proceed
9. The next window will prompt the user to get an image to determine which cell body processing method and strictness will work best
    a. The default setting of "Segmentation" with strictness of 5 is optimized to work with most images
        i. Dense cells require *lower* strictness for segmentation and *higher* strictness for thresholding
        ii. Strictness values are optimized to fall between 1 and 10, however, any non-zero value will be accepted but may result in reduced processing quality
    b. Select "Get image" and choose one of the sample images
    c. The macro will automatically process this image in 3 different strictness for both thresholding and segmentation types of cell body processing
    d. This may take a minute
    e. The macro will display the results of each processing method and strictness
        i. The best one will be the image that has each cell in its own ROI
        ii. ROIs without a nucleus will not be included in the analysis, however, excessive segmentation may slow processing speed
        iii. Note the best method and strictness from the image titles
    f. Either continue cycling through images to adjust threshold or, when satisfied, select "Next step (settings)" to continue to the main settings window

<u>AggreCount main settings window</u>

The main settings window will allow users to adjust the parameters described below. Some of these parameters may be populated from performing the setup. Settings are reset to default settings each time the macro is run. Settings from previous runs are saved in the "aggresettings.txt" file within the AC_analysis folder if "Save results" is checked.

1. Aggregate thresholding lower/upper
    a. The threshold values will be auto-populated from the user-selected threshold during setup
    b. This threshold values may be adjusted in this window
2. Perinuclear distance cutoff
    a. The distance from the nuclear perimeter that defines the perinuclear zone measured in pixels
3. Aggresome min size
    a. The size cutoff for designating a perinuclear aggregate an aggresome
4. Aggregate min/max size
    a. Size cutoff for aggregate ROI acquisition
    b. Aggregate ROIs smaller or larger than these values will not be captured
5. Nuclei size/strictness
    a. Minimum size of nuclei (helps exclude noise)
    b. Strictness defines the amount of background subtraction during nuclei processing
        i. Lower values *decrease* background subtraction, higher values *increase* it
6. Cell size/strictness
    a. Minimum size of cell ROIs (helps to exclude noise)
    b. Strictness alters cell body processing depending on the method

        i. Thresholding strictness increases or decreases background subtraction in the same manner as nuclei strictness
        ii. Segmentation strictness defines the "prominence" value in the "Find maxima…" function
            1. Smaller strictness values will increase total number of segments while larger values to decrease it
            2. Cell segments are checked for nuclei and segments without nuclei are deleted
7. Nuclei/aggregates/Cell bodies channel/slice/frame
    a. These fields are auto-populated from earlier in the setup process
    b. If the images do not have slices (z-stack) or frames (movie), these values should be 1
    c. If the images do not have a cell bodies channel, do not change these values and uncheck "Find cell bodies"
    d. NOTE – all images to be analyzed must have all channels in the same order
8. Find cell bodies checkbox
    a. If checked, the macro will find cell bodies using the processing method and strictness designated, aggregate distance will be calculated from the nucleus within the same cell and will output cell-by-cell data in the save file dataset_cells
    b. If unchecked, the macro will not find cell bodies, aggregate distance will be calculated from the closest nuclei, and will output image summary data and aggregate data but not single cell data
9. Cell processing method
    a. Segmentation uses the "Find maxima…" function in ImageJ to locate cell centers and then uses a watershed algorithm to create a Voronoi diagram. This is used to find cell ROIs which are checked for nuclei
    b. Thresholding uses the "Enhance contrast…" function to increase fluorescent signal, then adds in nuclei area before applying an automated threshold
10. Save results
    a. If checked, the macro will create a new folder in the image analysis folder previously selected named "AC_analysis#" where individual data files and ROI files as well as dataset files will be saved
    b. If unchecked, the macro will create tables in ImageJ with all data captured but will not save it
        i. The user may manually save this data if wanted
11. Batch mode
    a. If checked, the macro will proceed to analyze all images in the folder selected in the background without user input, greatly increasing processing speed
    b. If unchecked, the macro will prompt user before each image as well as display ROIs for nuclei, aggregates, and cells after capture
    c. It is suggested that users uncheck batch mode before running a batch analysis to ensure fidelity of ROI capture for a subset of images

<u>AggreCount analysis (non-batch mode)</u>

1. After proceeding through AC setup or manually entering AC settings, leave "Batch mode?" unchecked and press "OK"

2. For each file that is in the folder, the user will be prompted to analyze it (“Continue”), skip it (“Skip”), or quit the macro (“Cancel”)
    a. Non-image files or incompatible image files may appear but will be skipped even if the user selects “Continue”
3. After pressing “Continue”, nuclei will be processed, and an auto-threshold applied. The nuclei ROIs will be displayed for the user to view
    a. If nuclei ROIs are too large or there is excess noise, consider increasing nuclei processing strictness in the main menu
    b. If nuclei ROIs are too small or nuclei are being excluded, consider decrease nuclei processing strictness in the main menu
        i. To change nuclei processing strictness, quit out of the macro, run it again, and select “Manual input” to input desired settings
    c. Select “OK” to continue
4. Aggregates will be processed, and a threshold applied (as previously set by the user). The aggregate ROIs will be displayed for the user to view
    a. If aggregate thresholding requires further refinement, consider quitting the macro and restarting the setup process
    b. Select “OK” to continue
5. Cells will be processed as previously determined by the user (method and strictness). The cell ROIs will be displayed for the user to view
    a. Thresholding
        i. If ROIs are too large or multiple cells are captured in one ROI, consider increasing cell processing strictness
        ii. If ROIs are too small or cells are being excluded, consider decreasing cell processing strictness
    b. Segmentation
        i. If ROIs are too large and contain multiple cells, consider *decreasing* cell processing strictness
        ii. If ROIs are too small and are splitting cells, consider *increasing* cell processing strictness
    c. Select “OK” to continue
6. Aggregates and nuclei will be assigned to cells and aggregate localization is determined
    a. The dataset tables and the image data table will be updated with the values from the current image
7. The macro will pause after the entire analysis has been finished and will display the image along with all ROIs captured (nuclei, cell bodies and aggregates)
    a. Additionally, data will be presented in the “Data” table with cells and aggregates for the user to view
    b. Select “OK” to continue
8. The macro will again present files in the folder for the user to analyze or skip
    a. The user may proceed through all the images in this manner
    b. NOTE – the dataset tables will NOT save unless all files have been analyzed or skipped. To save these tables after canceling the macro run, select “File” -> “Save” on the table window.

<u>AggreCount analysis (batch mode)</u>

1. After proceeding through AC setup or manually entering AC settings, check “Batch mode?” and press “OK”
2. All images will be analyzed in the folder selected
3. Four tables will be visible to the user
    a. Dataset_summary
    b. Dataset_aggregates
    c. Dataset_cells (if “Find cells” is checked)
    d. Data
4. Each will be updated after each image is processed
5. Each line in Dataset_summary represents an image file so this table is ideal for tracking analysis process
6. WARNING – do not close these tables or select specific lines as it may interfere with the analysis
    a. These tables may be minimized
7. If the user wishes to cancel the batch mode analysis, close the Dataset tables
8. After the analysis is finished, the tables are saved to the “AC_analysis#” folder in the folder containing images

Analysis output

All data files are saved as tab-delimited .txt files. These may be easily imported into excel or another data processing software. In addition to the data tables described below, ROIs for each image are saved as .zip files within the AC_analysis folder. Each ROI zip file contains ROIs for cells, nuclei, and aggregates in that order.

Dataset_summary

Summary data by image

- File – Filename
- Cells – Number of cells imaged (uses # of nuclei)
- Aggregates per cell – Total aggregates in image divided by total number of cells (nuclei)
- Aggregate area per cell – Total aggregate area in image divided by total number of cells
- Avg aggregate size – Total aggregate area divided by total aggregates
- Cells with aggregates* – Total number of cells with any type of aggregate
- % cells with aggregates* – Percentage of all cells with any type of aggregate
- Cells with aggresome* – Total number of cells with an aggregate above the user defined size cutoff within the perinuclear zone (aggresome)
- % cells with aggresome* – Percentage of all cells with an aggregate above the user defined size cutoff within the perinuclear zone (aggresome)
- Total aggresomes** - Total number of aggresomes in the image
- % aggs perinuclear – Perinuclear aggregates divided by total aggregates
- % aggs cytosolic – Cytosolic aggregates divided by total aggregates
- % aggs nuclear – Nuclear aggregates divided by total aggregates
- Perinuclear aggregates per cell – Total perinuclear aggregates divided by total cells (nuclei)
- Perinuclear aggregate area per cell – Total perinuclear aggregate area divided by total cells (nuclei)
- Cytosolic aggregates per cell – Total cytosolic aggregates divided by total cells (nuclei)
- Cytosolic aggregate area per cell – Total cytosolic aggregate area divided by total cells (nuclei)

- Nuclear aggregates per cell – Total nuclear aggregates divided by total cells (nuclei)
- Nuclear aggregate area per cell – Total nuclear aggregate area divided by total cells (nuclei)

Dataset_cells*

Summary data by cell

- File – Filename of the image that contains the cell
- Nuclei – Number of nuclei contained within a cell ROI
- Aggregates – Total number of aggregates within a cell
- Aggregate area – Total aggregate area within a cell (um^2)
- Avg aggregate size – Total aggregates within the cell divided by total aggregate area within the cell
- Cytosolic aggregates – Total number of cytosolic aggregates within a cell
- Cytosolic agg area – Total cytosolic aggregate area within a cell
- Avg cyt agg size – Average cytosolic aggregate size within a cell
- Perinuclear aggregates – Total number of perinuclear aggregates within a cell
- Perinuclear agg area – Total perinuclear aggregate area within a cell
- Avg cyt agg size – Average cytosolic aggregate size within a cell
- Nuclear aggregates – Total number of nuclear aggregates within a cell
- Nuclear agg area – Total nuclear aggregate area within a cell
- Avg cyt agg size – Average cytosolic aggregate size within a cell
- Largest perinuclear agg – Size of the largest perinuclear aggregate within the perinuclear zone
- Area – Total area of a cell ROI
- Mean – Mean fluorescent intensity from a cell ROI
- StDev – Standard deviation of fluorescent intensity from a cell ROI

Dataset_aggregates

Summary data by aggregate

- File – Filename of image that contains the aggregate
- Location – Cellular compartment of an aggregate (nuclear, perinuclear, cytosolic)
- Distance – Distance in pixels between the nearest edge of an aggregate to a nucleus ROI
- Area – Area of the aggregate
- Mean – Mean fluorescent intensity from aggregate ROI
- StDev – Standard deviation of the fluorescent intensity from aggregate ROI

'Image name'_analysis

Summary data for a specific image. Contains data for both cell and aggregate ROIs

- Class – Classification of ROI: cell, aggregate, aggresome
- Cell # - Cell ID that links aggregates to cells
- Location – Subcellular location of aggregates (cytosolic, perinuclear, nuclear). Cell ROIs will be labeled "NA"
- Area – Area of a ROI
- Mean – Mean fluorescence intensity of a ROI
- StDev – Standard deviation of fluorescence intensity of a ROI

- Nuclei* – Number of nuclei contained within a cell ROI
  - Aggregate ROIs will have “0” in this column
- Aggregates* – Total number of aggregates in a cell
  - Aggregate ROIs will have “0” in this column
- Aggregate area* – Total sum of aggregate area in a cell
  - Aggregate ROIs will have “0” in this column
- Cytosolic agg* – Total number of cytosolic aggregates in a cell
  - Aggregate ROIs will have “0” in this column
- Cytosolic agg area* – Total sum of cytosolic aggregate area in a cell
  - Aggregate ROIs will have “0” in this column
- Perinuclear agg* – Total number of perinuclear aggregates in cell
  - Aggregate ROIs will have “0” in this column
- Perinuclear agg area* – Total sum of perinuclear aggregate area in a cell
  - Aggregate ROIs will have “0” in this column
- Nuclear agg* – Total number of nuclear aggregates in a cell
  - Aggregate ROIs will have “0” in this column
- Nuclear agg area* – Total sum of nuclear aggregate area in a cell
  - Aggregate ROIs will have “0” in this column
- Aggresomes* – Total number of aggresomes in a cell
  - Aggregate ROIs will have “0” in this column
- Aggresome area* – Total aggresome area in a cell
  - Aggregate ROIs will have “0” in this column
- Distance – Aggregate distance in pixels from a nucleus ROI perimeter
  - Cell ROIs will have “0” in this column

*designates fields or data tables that are only present in analyses that use cell ROIs

** designates fields that are only present in analyses that do not use cell ROIs

Supporting information 4

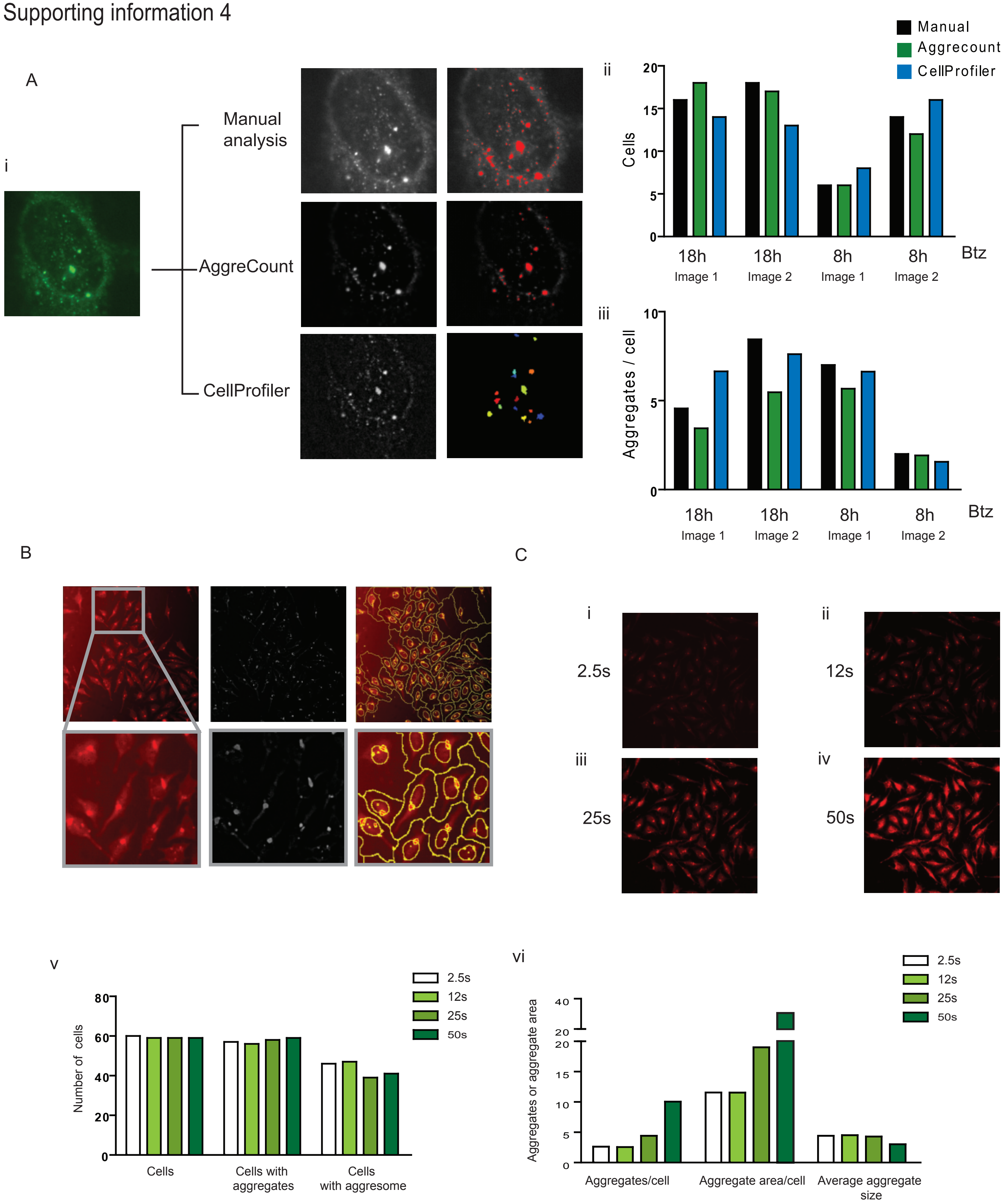

Supplement: Supporting Information [file supp_RA120.015398_162506_2_supp_613854_qwgfbm.pdf]
